# Hippocampal gamma and sharp-wave ripple oscillations are altered in a *Cntnap2* mouse model of autism spectrum disorder

**DOI:** 10.1016/j.celrep.2021.109970

**Published:** 2021-11-09

**Authors:** Rosalia Paterno, Joseane Righes Marafiga, Harrison Ramsay, Tina Li, Kathryn A. Salvati, Scott C. Baraban

**Affiliations:** 1 Department of Neurological Surgery and Weill Institute of Neuroscience, University of California, San Francisco, CA 94143, USA; 2 Neurophysiology and Neurochemistry of Neuronal Excitability and Synaptic Plasticity Laboratory, Graduate Program in Biological Sciences: Biochemistry, Department of Biochemistry, ICBS, Universidade Federal do Rio Grande do Sul, Porto Alegre, 90035-003, Brazil; 3 Lead contact

## Abstract

Impaired synaptic neurotransmission may underly circuit alterations contributing to behavioral autism spectrum disorder (ASD) phenotypes. A critical component of impairments reported in somatosensory and prefrontal cortex of ASD mouse models are parvalbumin (PV)-expressing fast-spiking interneurons. However, it remains unknown whether PV interneurons mediating hippocampal networks crucial to navigation and memory processing are similarly impaired. Using PV-labeled transgenic mice, a battery of behavioral assays, *in vitro* patch-clamp electrophysiology, and *in vivo* 32-channel silicon probe local field potential recordings, we address this question in a *Cntnap2*-null mutant mouse model representing a human ASD risk factor gene. *Cntnap2*^‒*/*‒^ mice show a reduction in hippocampal PV interneuron density, reduced inhibitory input to CA1 pyramidal cells, deficits in spatial discrimination ability, and frequency-dependent circuit changes within the hippocampus, including alterations in gamma oscillations, sharp-wave ripples, and theta-gamma modulation. Our findings highlight hippocampal involvement in ASD and implicate interneurons as a potential therapeutical target.

## In brief

Paterno et al. show that deletion of autism-associated Cntnap2 alters hippocampal PV+ interneuronal density and perisomatic inhibitory neurotransmission. Perturbed gamma oscillations and sharp wave ripples (SWP-Rs) in area CA1 of Cntnap2 KO mice associated with impaired spatial discrimination highlight the involvement of the hippocampus in the autism phenotype.

## Graphical abstract

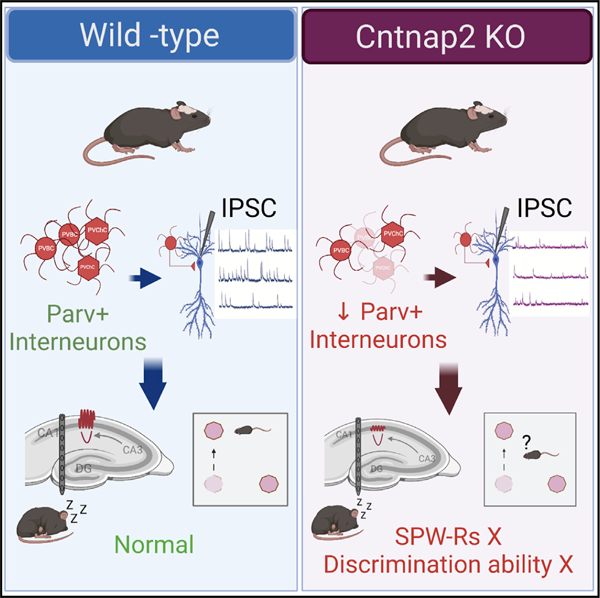

## INTRODUCTION

The incidence of autism spectrum disorder (ASD), a heterogenous condition characterized by hyperactivity, deficits in social interaction, and repetitive patterns of behavior, has risen dramatically over the past five decades (Taylor et al., 2020). Initially reported as 1 in 10,000 cases, ASD incidence is now 2 in 100 cases (Kim et al., 2011; Baio et al., 2018; Schendel and Thorsteinsson, 2018). Genome-wide association studies on these patients indicate a genetic basis spanning hundreds of different risk genes (Abrahams et al., 2013; Iakoucheva et al., 2019). As a neurodevelopmental disorder diagnosed as early as 2 years of age (Hyman et al., 2020), ASD significantly impacts neural circuits across many brain regions including the cerebellum, somatosensory cortex, and prefrontal cortex (Golden et al., 2018). Within these brain regions, a common simplified model is that excitatory/ inhibitory (E/I) imbalance, presumably linked to GABAergic interneuron dysfunction, represents a neurophysiological hallmark of ASD (Rubenstein and Merzenich, 2003; Gogolla et al., 2009; Lee et al., 2017; Sohal and Rubenstein, 2019). Consistent with this hypothesis, postmortem human ASD studies describe: (1) reduced interneuron density (Ariza et al., 2018; Hashemi et al., 2018), (2) decreased GABA receptor subunit expression (Blatt et al., 2001; Guptill et al., 2007; Fatemi et al., 2010), and (3) decreased magnetic resonance (MR) spectroscopy-measured GABA levels (Harada et al., 2011). Recapitulating human genetic mutations associated with ASD, mouse models further support a GABAergic interneuron dysfunction hypothesis (Chao et al., 2010; Vogt et al., 2015; Selimbeyoglu et al., 2017) and specifically highlight alterations in parvalbumin (PV)-positive fast-spiking interneurons in *Shank3* (Filice, 2016), *Mecp2* (Ito-Ishida et al., 2015), and *Cntnap2* (Peñagarikano et al., 2011; Vogt et al., 2018) mutant mice as well as an environmental-induced ASD model such as prenatal valproate exposure (Lauber et al., 2016).

In the hippocampus, a PV-positive interneuron subpopulation, which includes further subtypes such as basket cells, axoaxonic cells, and bistratified neurons (Que et al., 2021), primarily innervate the soma and proximal dendrites of excitatory pyramidal neurons (Miles et al., 1996). These neurons represent a subset (~25%) of the total interneuron population (Kosaka et al., 1987), derive from progenitor cells in a subpallial embryonic region designated the medial ganglionic eminence (MGE) (Gelman and Marín, 2010), and can make connections with up to 1,000 pyramidal cells (Bezaire and Soltesz, 2013; Li et al., 1992). Hippocampal PV-positive interneurons play a fundamental role in the generation and maintenance of network oscillations associated with learning and episodic memory (Cardin et al., 2009; Sohal et al., 2009). For example, hippocampal PV-positive cells resonating at gamma frequency (40–120 Hz) exert perisomatic inhibition onto CA1 pyramidal cells to control spike generation (Cobb et al., 1995; Miles et al., 1996) and spike timing (Fernández-Ruiz et al., 2017) necessary for the functional processes underlying memory formation or spatial navigation (Burgess, 2008; Hasselmo et al., 2002). Hippocampal PV-positive interneurons are also essential for timing and spatial pyramidal cell synchrony during sharp wave ripples (SWRs) thought to play a role during navigation and memory consolidation (Buzsáki, 2015). Indeed, numerous studies suggest that PV-positive basket cell activity is phase-locked to ripple cycles (Ylinen et al., 1995; Klausberger et al., 2003; Rácz et al., 2009; Varga et al., 2012), and more importantly, activation of these interneurons can induce coherent ensemble spiking in pyramidal cells (Stark et al., 2014). Critically, these studies suggest an essential role for a sub-class of interneurons (e.g., PV-expressing fast-spiking interneurons) in dynamically regulating the E/I balance during normal network processing. Given these findings, one would suspect that hippocampal PV-positive interneuron deficit could result in a significant disruption of hippocampal-dependent network activity. While inhibitory interneuron density deficits have been reported (Ariza et al., 2018; Hashemi et al., 2018), a contrasting immunohistochemistry study in hippocampal tissue from 5 ASD patients described increased PV+ interneuron density (Lawrence et al., 2010). Because a systematic evaluation of hippocampal interneuron function in human ASD patients is not feasible, we focused our efforts on an established mouse model of the disease.

Among identified human ASD risk genes, contactin-associated protein-like 2 (*CNTNAP2*), which encodes a synaptic cell adhesion molecule, is considered by the Simons Foundation Autism Research Initiative (SFARI) to be a strong ASD candidate. Indeed, a loss-of-function *CNTNAP2* gene mutation was identified in a cohort of ASD patients (Strauss et al., 2006), and *CNTNAP2* polymorphism has been associated with an increased risk of ASD (Arking et al., 2008). *Cntnap2* expression in mouse MGE at embryonic day 13.5 (E13.5) suggests a role in development/maturation of somatostatin (SOM)- and parvalbumin (PV)-expressing interneurons (Gordon et al., 2016; Vogt et al., 2018). Indeed, null *Cntnap2* mutant mice have a modest, but significant, reduction of PV-positive cells in somatosensory cortex, striatum, and hippocampus (Peñagarikano et al., 2011; Lauber et al., 2018; Vogt et al., 2018). These mice also replicate clinical phenotypes including epileptiform discharges, hyperactivity, social interaction deficits, and repetitive behavior (Peñagarikano et al., 2011). While considerable work has been done to examine the functional consequences of *Cntnap2* mutation on cortical circuits and associated behaviors, much less is known about how *Cntnap2* regulates hippocampal networks and hippocampal-dependent behaviors.

To investigate the role of *Cntnap2* in the hippocampus, we performed a comprehensive series of anatomical, behavioral, *ex vivo,* and *in vivo* electrophysiological studies using adult *Cntnap2* knockout (KO) mice. We found a reduction in PV-labeled interneuron density in area CA1 and diminished synaptic inhibition onto CA1 pyramidal cells. Using 32-channel silicon probes, we uncovered frequency-dependent circuit changes with specific alterations in gamma oscillations (65–90 Hz), sharp-wave ripples (150–250 Hz), and theta-gamma modulation in area CA1. At a behavioral level, *Cntnap2* KO mice were characterized by hyperactivity and deficits in hippocampal-dependent spatial object recognition in addition to the already established behavioral phenotypes such as repetitive behavior and altered levels of anxiety. Taken together, these findings identify a role for *Cntnap2*, particularly in hippocampal PV+ interneurons, and provide insights into ASD-associated behaviors.

## RESULTS

### PV interneuron density is reduced in the hippocampus of Cntnap2 KO mice

**Figure 1.**
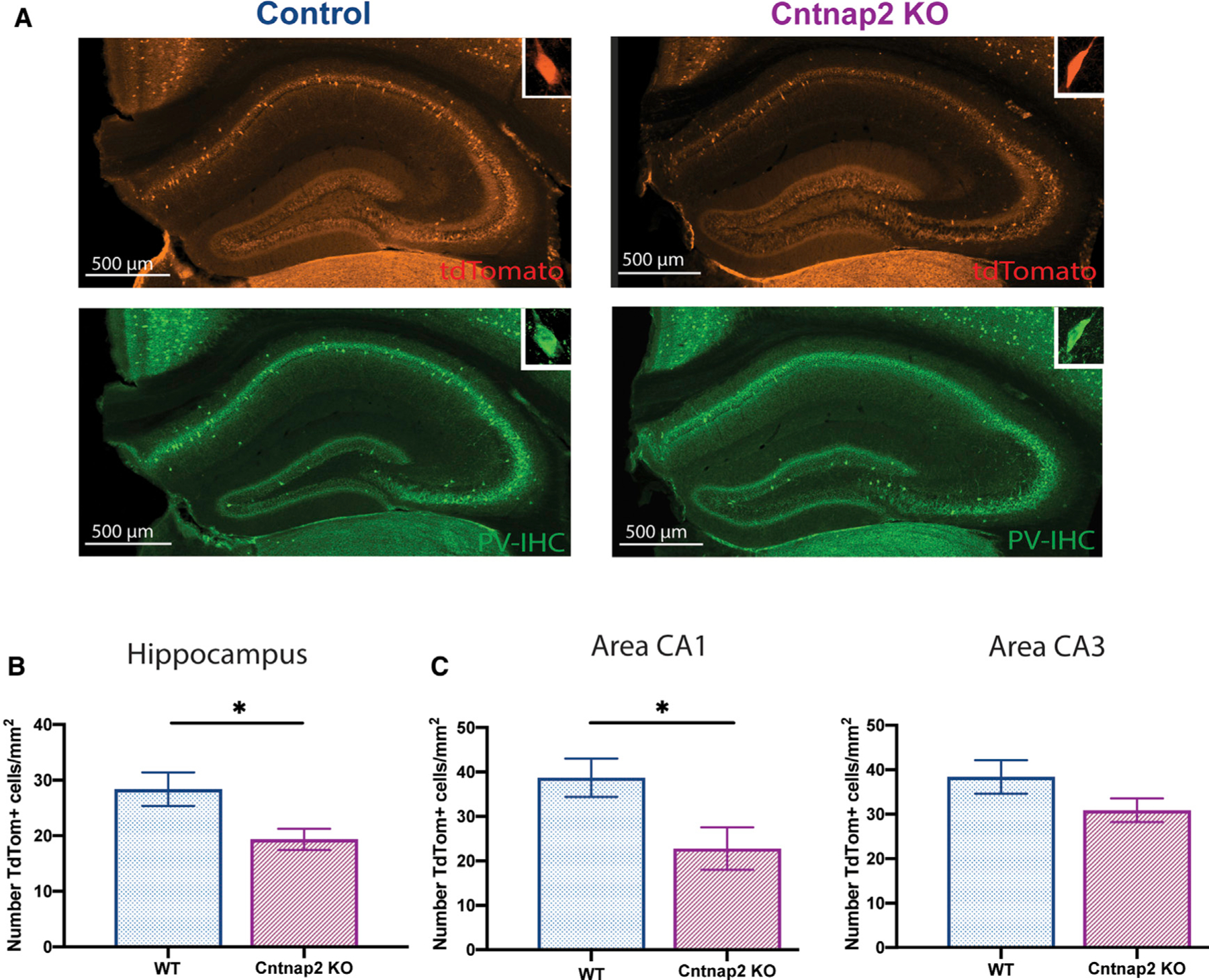
Reduced number of PV^+^ interneurons (INs) in the hippocampus (A) Example of hippocampal coronal section from WT PV tdTomato (TdT)^(+/‒)^ and *Cntnap2* KO PV TdT^(+/‒)^ mice. (B) Distribution of TdT-positive cells in the hippocampus (WT: 28 ± 3 cells/mm^2^, n = 6 mice; Cntnap2 KO: 19 ± 4 cells/mm^2^, n = 4 mice; unpaired t test, t (2.5), p = 0.03). (C) Left panel: distribution of TdT-positive cells in area CA1 (WT: 38 ± 4 cells/mm^2^, n = 6 mice; *Cntnap2* KO: 23 ± 5 cells/mm^2^, n = 4 mice; unpaired t test, t (2.5), p = 0.04). Right panel: area CA3 (WT: 38 ± 4 cells/mm^2^, n = 6 mice; *Cntnap2* KO: 30 ± 3 cells/mm^2^, n = 4 mice; unpaired t test, t (1.6), p = 0.1). Data are presented as mean ± SEM.

Interneuron deficits have been reported in the prefrontal cortex of ASD patients (Ariza et al., 2018; Hashemi et al., 2018) and several genetic mouse models of ASD including *Cntnap2* KO mice (Gogolla et al., 2009; Vogt et al., 2018). Additionally, in the *Cntnap2* KO animal model, decreased PV+ interneuron density was described in striatum, somatosensory cortex, and hippocampus (Peñagarikano et al., 2011; Lauber et al., 2018); however, other studies failed to report differences in PV+ interneuron density in mPFC (Lazaro et al., 2019) or hippocampus (Lauber et al., 2018). To address this discrepancy in PV+ interneuron density in hippocampus, we crossed *Cntnap2* mice with a transgenic Pvalb-tdTomato reporter line (Jackson laboratory #027395). In hippocampal sections from adult wild-type (WT; 6 coronal sections; n = 2 mice) mice, we first confirmed that 91% of Pvalb-tdTomato fluorescent cells co-labeled with an antibody-recognizing PV (Figure 1). Next, using a blinded stereological assessment of tdTomato-fluorescent cells, we confirmed a reduction in overall density of PV+ interneurons for *Cntnap2* KO mice compared to age-matched WT controls (Figure 1B). Parsing these cell counts by hippocampal sub-region, we noted that reduced PV+ interneuron density was only significant in area CA1 (Figure 1C, left panel). These data indicate a role for *Cntnap2* in development or maturation of PV-positive hippocampal interneurons.

### Decreased inhibitory synaptic transmission in CA1 pyramidal neurons from Cntnap2 KO mice

**Figure 2.**
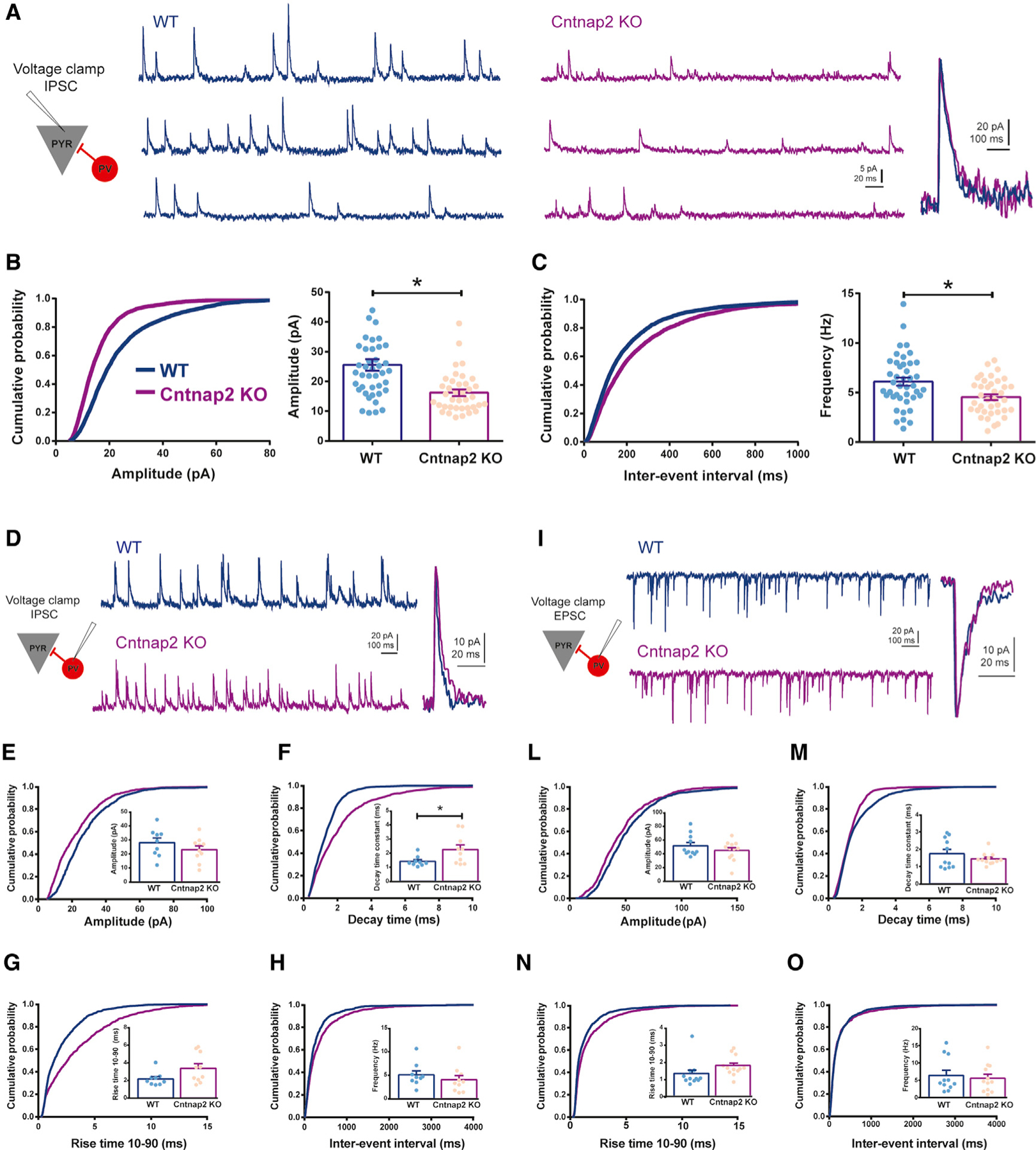
Decreased inhibition in CA1 pyramidal neurons in Cntnap2 KO mice (A) Representative traces of IPSCs recorded from CA1 pyramidal neurons in WT and *Cntnap2* KO mice, voltage-clamped at 0 mV, with normalized unitary events (left) demonstrating no differences in the kinetics of IPSCs. (B) Cumulative distribution and average of IPSC amplitude recorded from pyramidal neurons (WT: 24.24 ± 1.51 pA, n = 41, 7 mice; *Cntnap2* KO: 16.22 ± 1.09 pA, n = 38, 8 mice; two-tailed unpaired t test, t (4.22), p < 0.0001; cumulative distribution, Kolmogorov-Smirnof [K-S] test: WT versus KO p < 0.0001). (C) Cumulative distribution and average plots of IPSC frequency recorded from pyramidal neurons (WT: 6.13 ± 0.41 Hz, n = 41, 7 mice; *Cntnap2* KO: 4.54 ± 0.29 Hz, n = 38, 8 mice; two-tailed unpaired t test, t (3.08), p = 0.0029; cumulative distribution, K-S test: WT versus KO p < 0.0001). Note a decreased IPSC amplitude and frequency in pyramidal neurons in *Cntnap2* KO mice. (D–H) Whole-cell patch-clamp IPSC recording from PV+ interneurons located in CA1 pyramidal cell layer in WT and Cntnap2 *KO* mice. (D) Representative traces of IPSCs voltage-clamped at 0 mV, with corresponding normalized unitary events in WT and *Cntnap2* KO mice. (E) IPSC amplitude (WT: 28.02 ± 3.33 pA, n = 9, 7 mice; *Cntnap2* KO: 23.07 ± 2.70 pA, n = 10, 6 mice; two-tailed unpaired t test, t (1.16), p = 0.2612; cumulative distribution, K-S test: WT versus KO p < 0.0001). (F) IPSC decay time constant (WT: 1.41 ± 0.12 ms, n = 9, 7 mice; *Cntnap2* KO: 2.249 ± 0.32 ms, n = 10, 6 mice; two-tailed unpaired t test, t (2.310), p = 0.0337; cumulative distribution, K-S test: WT versus KO p < 0.0001). (G) IPSC rise time 10–90 (WT: 2.16 ± 0.26 ms, n = 9, 7 mice; *Cntnap2* KO: 3.36 ± 0.52 ms, n = 10, 6 mice; two-tailed unpaired t test, t (1.982), p = 0.0638; cumulative distribution, K-S test: WT versus KO p < 0.0001). (H) IPSC frequency (WT: 5.06 ± 0.83 Hz, n = 9, 7 mice; *Cntnap2* KO: 4.02 ± 0.90 Hz, n = 10, 6 mice; two-tailed unpaired t test, t (0.84), p = 0.41; cumulative distribution, K-S test: WT versus KO p < 0.0001). (I–O) Whole-cell patch-clamp EPSC recording from PV+ interneurons located in CA1 pyramidal cell layer in WT and *Cntnap2* KO mice. (I) Representative traces of EPSCs voltage-clamped at ‒65 mV, with corresponding normalized unitary events in WT and *Cntnap2* KO mice. (L) EPSC amplitude (WT: 51.76 ± 4.73 pA, n = 11, 7 mice; *Cntnap2* KO: 44.87 ± 4.13 pA, n = 12, 6 mice; two-tailed unpaired t test, t (1.102), p = 0.2831; cumulative distribution, K-S test: WT versus KO p < 0.0001). (M) EPSC decay time constant (WT: 1.73 ± 0.25 ms, n = 11, 7 mice; *Cntnap2* KO: 1.43 ± 0.099 ms, n = 12, 6 mice; two-tailed unpaired t test, t (1.123), p = 0.2742; cumulative distribution, K-S test: WT versus KO p < 0.0001). (N) EPSC rise time 10–90 (WT: 1.33 ± 0.23 ms, n = 11, 7 mice; *Cntnap2* KO: 1.79 ± 0.16 ms, n = 12, 6 mice; two-tailed unpaired t test, t (1.639), p = 0.1162; cumulative distribution, K-S test: WT versus KO p < 0.0001). (O) EPSC frequency (WT: 6.38 ± 1.50 Hz, n = 11, 7 mice; *Cntnap2* KO: 5.55 ± 1.17 Hz, n = 12, 6 mice; two-tailed unpaired t test, t (0.44), p = 0.66; cumulative distribution, K-S test: WT versus KO p = 0.87). Results are expressed as mean ± SEM. IPSCs, inhibitory postsynaptic currents; EPSCs, excitatory postsynaptic currents. Data are presented as mean ± SEM.

Impaired perisomatic inhibition may be a common pathophysiological alteration in ASD, including *Cntnap2* KO mice (Gogolla et al., 2009; Jurgensen and Castillo, 2015). Because PV+ interneurons play a key role in mediating perisomatic inhibition (Freund and Katona, 2007) and are reduced in the CA1 region of adult *Cntnap2* KO mice (Figure 1), we examined inhibitory transmission in acute hippocampal slices using visualized whole-cell patch-clamp recording techniques. We measured spontaneous inhibitory postsynaptic currents (sIPSCs) in CA1 pyramidal cells as a standard measure of inhibition. Representative traces are shown in Figure 2A. We found decreased sIPSC amplitude and frequency (Figures 2B and 2C) in *Cntnap2* KO mice compared to WT age-matched controls; no change in sIPSC kinetics were noted (Table S1).

Because decreased inhibition could result from a change in synaptic drive onto soma-innervating inhibitory neurons, we next measured sIPSCs (Figure 2D) and excitatory postsynaptic currents (EPSCs; Figure 2I) onto tdTomato-labeled PV+ interneurons in the stratum pyramidale (str. pyramidale) of area CA1. While a small, but statistically significant, change in sIPSC decay time was noted (Figure 2F), measures of amplitude, rise-time, inter-event interval, and frequency were unchanged (Figures 2E and 2L–2O), suggesting that the overall synaptic drive onto PV+ interneurons in adult *Cntnap2* KO mice is normal. To test whether deletion of *Cntnap2* effected intrinsic firing properties of PV+ interneurons, we measured resting membrane potential, input resistance, action potential threshold, frequency, amplitude, and half-width from td-Tomato-labeled cells; no changes were noted (Figure S3; Table S2). These results confirm an impairment of inhibitory neurotransmission in the CA1 region of hippocampus that is consistent with reduced PV+ interneuron density (Figure 1).

### Hippocampal-dependent spatial memory performance is impaired in adult Cntnap2 KO mice

**Figure 3.**
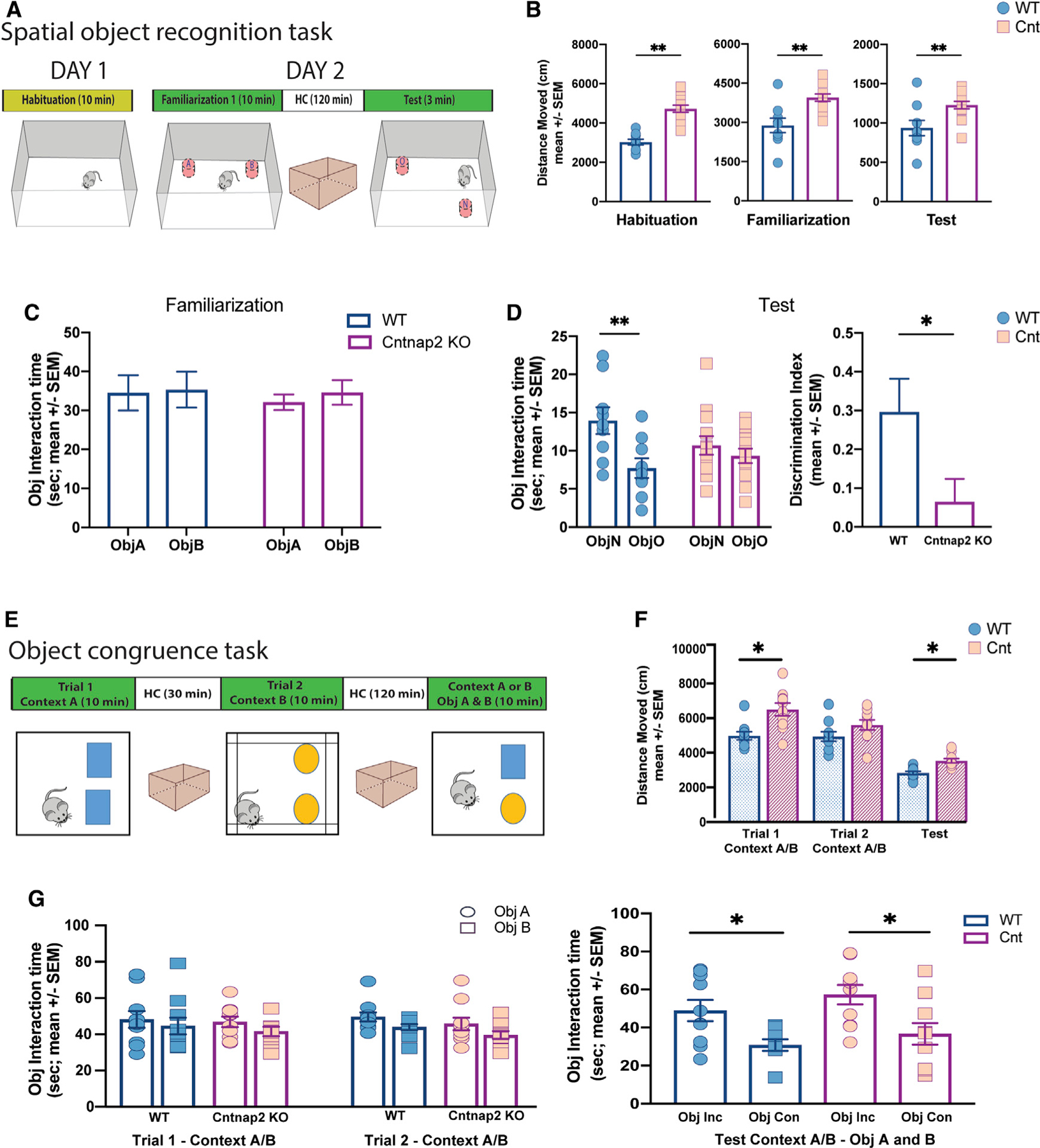
Spatial object recognition memory compromised in Cntnap2 KO mice (A) Schematic representation of the one-trial spatial object recognition task. The scheme displays the sequence of the trials across days and the position of the objects in the task during familiarization and test. (B) Total exploratory distance during habituation (10 min), familiarization (10 min), and test (3 min) in control (n = 9) and *Cntnap2 KO* (n = 13) mice. Note the hyperactive performance in *Cntnap2 KO* mice across all trials. Unpaired t test (habituation), t (20), p < 0.001. Unpaired t test (familiarization), t (12.1), p < 0.01. Unpaired t test (test), t (11.9), p < 0.03. (C) Cumulative time exploring objects during familiarization (ANOVA p > 0.05). (D) Left panel: cumulative time exploring objects during test (ANOVA p < 0.05; Sidak’s multiple comparisons test, WT object [Obj] A versus Obj B: p < 0.001, Cntnap2 KO Obj A versus Obj B: p = 0.6). Right panel: discrimination Index (DI) in WT and Cntnap2 KO during test. Unpaired t test, t (15.3), p < 0.05. Note that *Cntnap2 KO* were not able to discriminate the object moved in the new location. (E) Schematic representation of the object congruence task. The scheme displays the different contexts and the objects features and location in the task. (F) Total exploratory distance across trials in control (n = 10) and *Cntnap2* KO (n = 10) mice. Unpaired t test trial 1, t (15.1), p < 0.01; unpaired t test trial 2, t (17.9), p = 0.1; unpaired t test test, t (17), p < 0.01. Note that *Cntnap2* KO showed hyperactivity during task performance. (G) Left: Cumulative time exploring objects during trials 1 and 2. (ANOVA p > 0.05). Right: Cumulative time exploring objects during test (ANOVA p < 0.05; Sidak’s multiple comparisons test, WT Obj congruent versus Obj non-congruent: p < 0.03; *Cntnap2* KO Obj congruent versus Obj non-congruent: p < 0.01). Note that both animal conditions, WT and *Cntnap2* KO, were able to discriminate the object not congruent with the context. Data are presented as mean ± SEM.

Given the essential contribution of hippocampus to memory and navigation (O’Keefe and Nadel, 1978), we next explored potential behavioral deficits in *Cntnap2* KO mice using a hippocampal-dependent one-trial spatial object location task (SOR) (Figure 3A). As previously reported (Peñagarikano et al., 2011), *Cntnap2* KO mice exhibit generalized hyperactivity, quantified as increased distance moved across all trials of the task (habituation, familiarization, and test; Figure 3B). Despite this hyperactivity, *Cntnap2* KO mice showed similar object interaction times during familiarization compared to age-matched WT controls (Figure 3C). During test, however, *Cntnap2* KO animals exhibit impaired discrimination ability between the object located in the new versus the old location (Figure 3D).

To determine whether *Cntnap2* KO animals had a general deficit in object-context association, we implemented the object congruence task. During this task, also referred to as a ‘‘pattern separation task’’ (Figure 3E), *Cntnap2* KO mice displayed overall hyperactivity during trial 1 (Figure 3F), but no differences were found in objects’ interaction time during trial 1, trial 2, or the test trial. The task performed showed that under both conditions, age-matched *Cntnap2* KO and WT mice can discriminate which objects were not congruent with the current context during the test. These findings suggest that both *Cntnap2* KO and WT mice spent more time exploring the incongruent object (or object previously presented in a different context), as shown in Figure 3G. Collectively, these results indicate that *Cntnap2* KO mice exhibit a specific spatial memory deficit.

To provide a more comprehensive evaluation of potential autism-related behavioral deficits, mice were also evaluated in an elevated plus maze (Figures S1A–S1D) (Walf and Frye, 2007), a modified 3-chamber social test (Figures S1E–S1H) (Yang et al., 2011), self-grooming (Figures S1I–S1L) (Kalueff et al., 2016), and open field assays (Figures S1M–S1O) (Seibenhener and Wooten, 2015). Deficits in the elevated plus maze, self-grooming, and the first 5 min of the open field test are congruent with previously published autism-like behavioral phenotypes described for *Cntnap2*-null (Peñagarikano et al., 2011) and other mouse models of ASD (Silverman et al., 2010). These results further validated hyperactivity, repetitive behavior, and decreased levels of anxiety in *Cntnap2* KO mice.

### Gamma activity disruption in a layer-specific distribution in Cntnap2 KO during open field navigation

**Figure 4.**
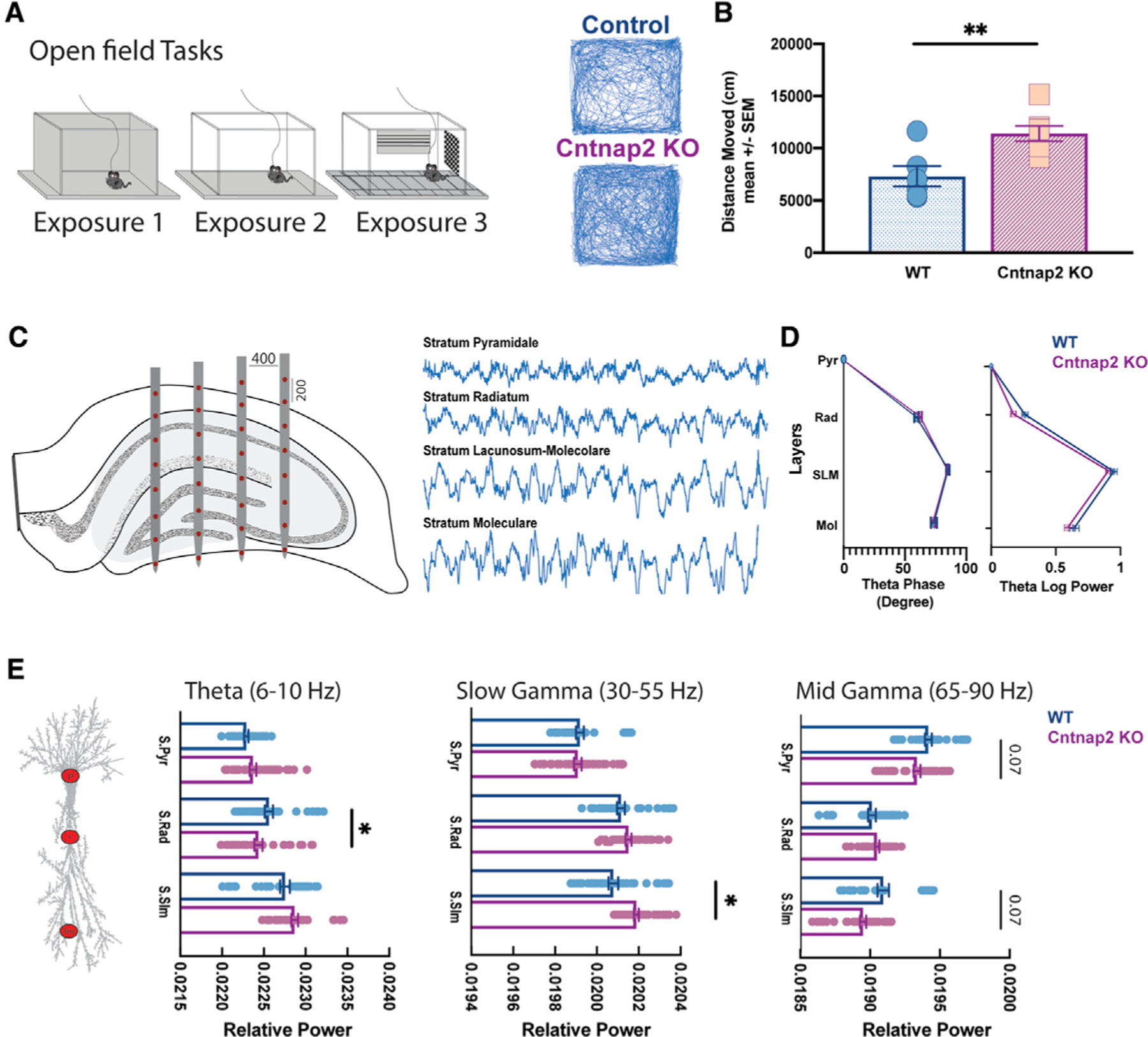
Power spectrum layer-specific changes during open field navigation (A) Left panel: schematic view of the open fields used in this experiment. The recording session was 25 min. Right panel: sample tracking during task exposure in WT (top) and *Cntnap2 KO* (bottom) (B) Averaged exploratory distance moved across animals. WT (6 animals): 7,313 cm; Cntnap2 KO (7 animals): 11,408 cm; unpaired t test, t (3.3), p < 0.01. (C) Example of the 32-channel electrode array implanted in the dorsal hippocampus and representative electrophysiological recording across the CA1-DG axis. (D) Layer shift of phase (left) and power (right) in theta rhythm in WT (6 animals) and *Cntnap2* KO (7 animals). Note the phase shift in str. LM and the higher theta power in str. LM in both conditions. (E) Relative power difference in theta (6–10 Hz), slow (25–55 Hz), and mid gamma (65–90 Hz) across CA1 hippocampal layers in WT and *Cntnap2* KO. Data are presented as mean ± SEM. *p < 0.05.

To investigate theta-gamma dynamics along the hippocampal axis, we recorded local field potentials (LFPs) from 32 channels arranged on a 4-shank silicon probe covering most layers of CA1, CA3, and DG (Figure S2) while the animal navigated an open field. Mice were exposed to different open field settings as shown in Figure 4A for 2 trials per day (25 min for each recording session). During each exposure, *Cntnap2* KO mice were more active compared to WT in the 3 different contexts (Figure 4B; Figure S4), further confirming an autism-like phenotype and persistence of these behavioral features after electrode implantation.

To isolate our analysis to theta periods during open field navigation, we restricted LFP analysis to theta epochs while animals were walking/running. Both groups ran at a similar speed during open field navigation (control n = 6; median speed 9.6 cm/s; *Cntnap2* KO n = 7; median speed 10.6 cm/s; unpaired t test with Welch correction p > 0.1). We first confirmed physiological features of the hippocampal anatomical layers using power and phase of theta rhythm along the hippocampal axis (Figure 4C). As expected (Buzsáki, 2002), we noticed a progressive theta phase shift along the axis, with maximal shift occurring in CA1 stratum lacunosum-moleculare (str. LM) in addition to the peak amplitude of the laminar theta power profile occurring in str. LM (Figure 4D). *Cntnap2* KO mice showed a similar layer-specific profile. To compare power across conditions, we calculated relative power in theta, slow, and mid gamma frequency ranges. In area CA1, *Cntnap2* KO mice exhibited a statistically significant decrease in theta (6–10 Hz) in str. radiatum, increased slow-gamma power (30–55 Hz) in str. LM, and a trending decrease in mid gamma power (65–90 Hz) in str. pyramidale and str. LM (Figure 4E). In the DG (str. moleculare), no differences in any frequency band examined (p > 0.1) (data not shown) were observed between control and *Cntnap2* KO mice.

### Cntnap2 KO exhibit a reduced mid gamma-theta phase modulation

**Figure 5.**
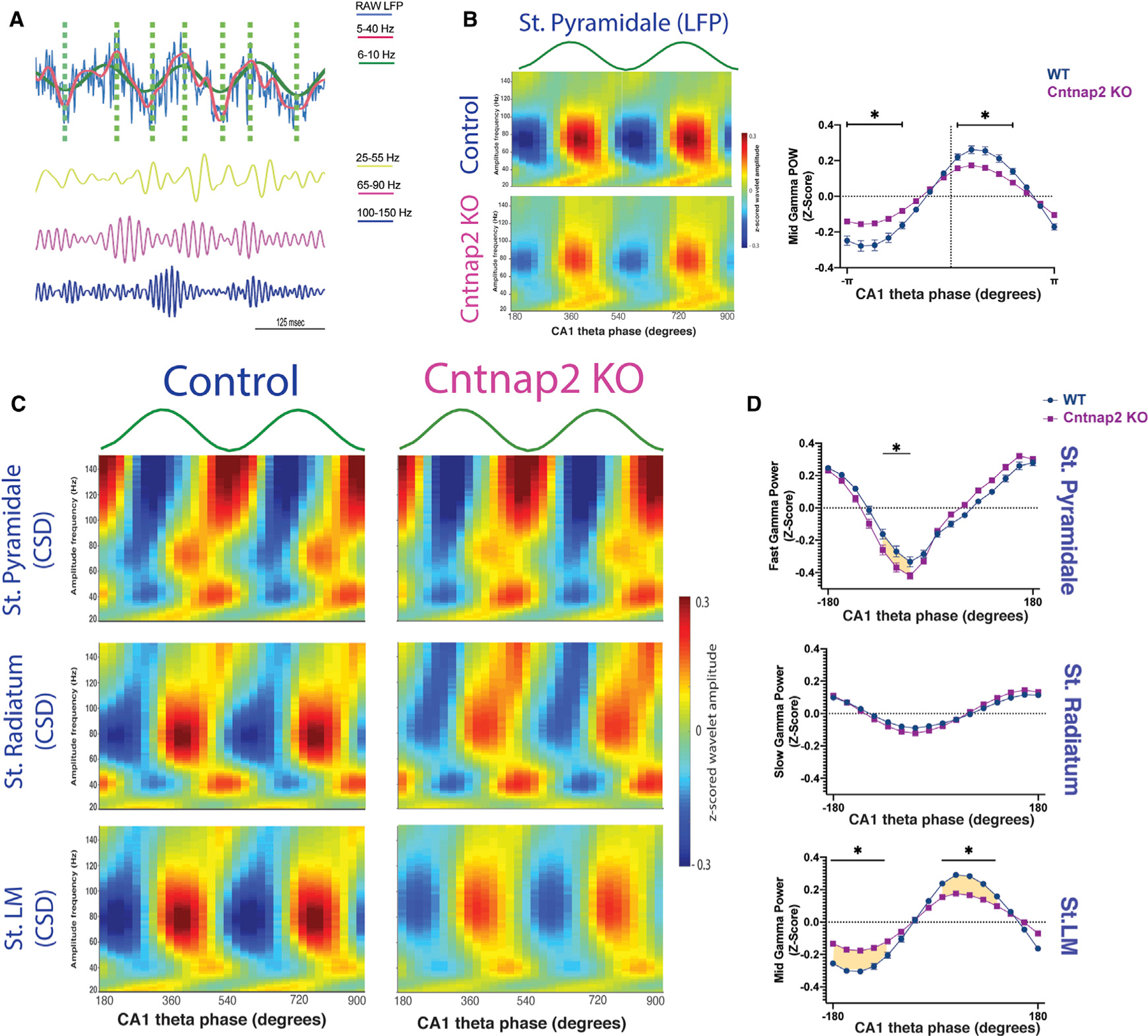
Reduced mid gamma-theta modulation in Cntnap2 KO (A) Example of theta cycles extraction. Top panel: the blue line represents the raw LFP, the red line shows the filtered signal at 5–40 Hz, and the green line represents the extracted theta signal. The vertical green dashed lines mark the maxima and minima of each theta cycle. Bottom panel: gamma-filtered activity from the LFP signal shown above in slow (yellow: 25–55 Hz), mid (pink: 65–90 Hz), and fast gamma (blue: 100–150 Hz). Note increased power in mid gamma at the peak and fast gamma at the trough of the theta cycles. (B) Left panel: gamma amplitude modulation by theta phase averaged across all theta cycles in each condition using LFP signal recorded in the str. pyramidale (WT, n = 6; *Cntnap2* KO, n = 7). Right panel: average mid gamma power (65–90 Hz) modulation by theta cycles phases across sessions (WT, n = 6, sessions: 36; *Cntnap2* KO, n = 7; sessions: 42). Multiple t test corrected using Holm-Sidak method. *p < 0.01. Note the significant reduced modulation at the peak of the theta cycle in *Cntnap2* KO mice. (C) Same analysis performed in (B) using CSD signal of the LFPs was able to isolate fast gamma in pyramidal cell layer, slow gamma in str. radiatum, and mid gamma in str. LM. (D) Averaged fast, slow, and mid gamma power modulated by theta phase across session in WT and *Cntnap2* KO mice in str. pyramidale, str. radiatum, and str. LM, respectively. Multiple t test corrected using Holm-Sidak method. *p < 0.03. Note the strong reduction of the modulation of mid gamma and theta phase in stratum lacunosum moleculare. Data are represented as mean ± SEM.

In area CA1 of the hippocampus, theta cycles, the most prominent oscillatory activity, are thought to be temporal windows in which different gamma bands transmit inputs from upstream regions. In particular, slow gamma (30–50 Hz), mid gamma (60–100 Hz), and fast gamma (>100 Hz) occur at descending, peak, and trough of the theta cycle (Colgin et al., 2009; Lasztóczi and Klausberger, 2014; Schomburg et al., 2014; Fernández-Ruiz et al., 2017). To determine whether loss-of-function of *Cntnap2* interrupts theta-gamma co-modulation, we next examined distribution of gamma oscillations over the phase of individual theta cycles (Figure 5A). As expected, for both *Cntnap2* KO and WT mice, slow gamma, mid gamma, and fast gamma dominated the descending, peak, and trough of the theta cycle, respectively (Figure 5B, left panel). However, modulation of power for mid gamma was significantly decreased at the peak of the theta cycle for *Cntnap2* KO mice (Figure 5B).

To identify the contribution of different CA1 layers to gamma band oscillations (Lasztóczi and Klausberger, 2014), we next implemented current source density (CSD) to spatially separate LFP signals. CSD signals isolated fast gamma from str. pyramidale, slow gamma from str. radiatum, and mid gamma from str. LM recordings, as shown in Figure 5C. *Cntnap2* KO mice exhibited increased modulation in fast gamma in pyramidal layers and a statistically significant decreased modulation in mid gamma in str. LM (Figure 5D). No differences were found in slow gamma-theta phase modulation in str. radiatum.

**Figure 6.**
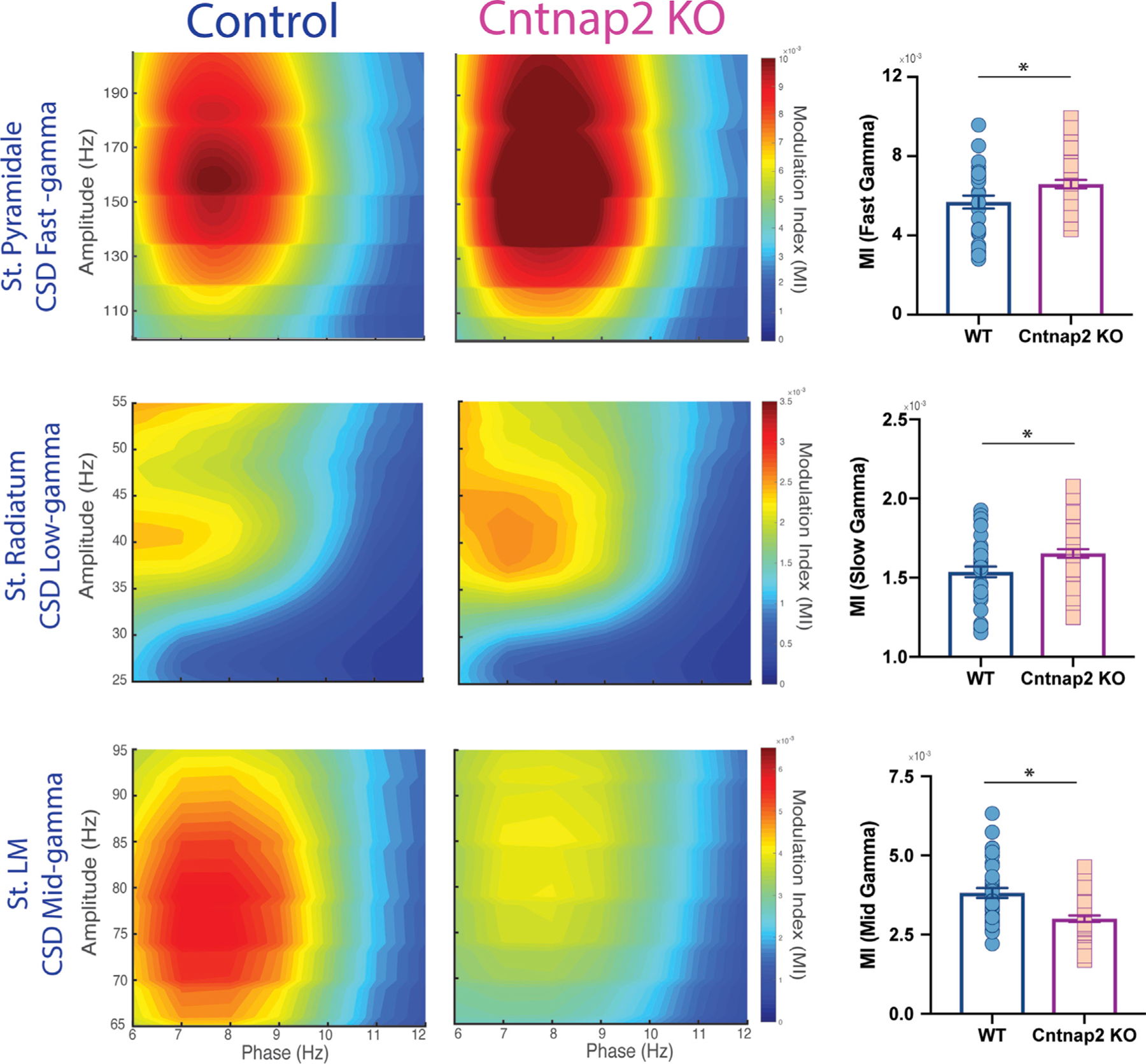
Phase-amplitude coupling defects in Cntnap2 KO mice Left panel: CSD signals were able to isolate phase-amplitude patterns in CA1 sublayers. CSD-derived LFP shows fast (>100 Hz) gamma-theta modulation from str. pyramidale and mid-gamma mid (65–90 Hz) gamma-theta modulation from str. LM. Right panel: group data of modulation index (MI) for fast gamma in str. pyramidale (WT, 6 session 36; *Cntnap2* KO, 7, 42 sessions, Mann-Whitney U test, p < 0.05), slow gamma in str. radiatum (WT, 6 session 36; *Cntnap2* KO, 7, 42 sessions; Mann-Whitney U test, p < 0.01), and mid gamma in str. LM across sessions (WT, 6 session 36; *Cntnap2* KO, 7, 42 sessions; Mann-Whitney U test, p < 0.001). Data are represented as mean ± SEM. Circles represent each recording session. *p < 0.05.

To further investigate the theta-gamma interaction, we calculated power modulation of high (20–200 Hz) frequency spectral activity as a function of the phase of theta oscillations (6–12 Hz). Cross-frequency coupling assessed as theta-gamma modulation index (MI) was performed in the CSD signals. As expected, in both conditions, the highest MI found was between theta and fast gamma (>120 Hz) in str. pyramidale and between theta and mid gamma (65–90 Hz) in str. LM. However, *Cntnap2* KO mice showed an increased MI between theta and fast gamma in CA1 pyramidal cell layers and between theta and slow gamma in str. radiatum and a decreased MI between theta and mid gamma in str. LM (Figure 6). Taken together, these findings suggest altered input processing in the different layers of hippocampal CA1 in *Cntnap2* KO mice.

### Decreased SWR power during immobility in home-cage recording

**Figure 7.**
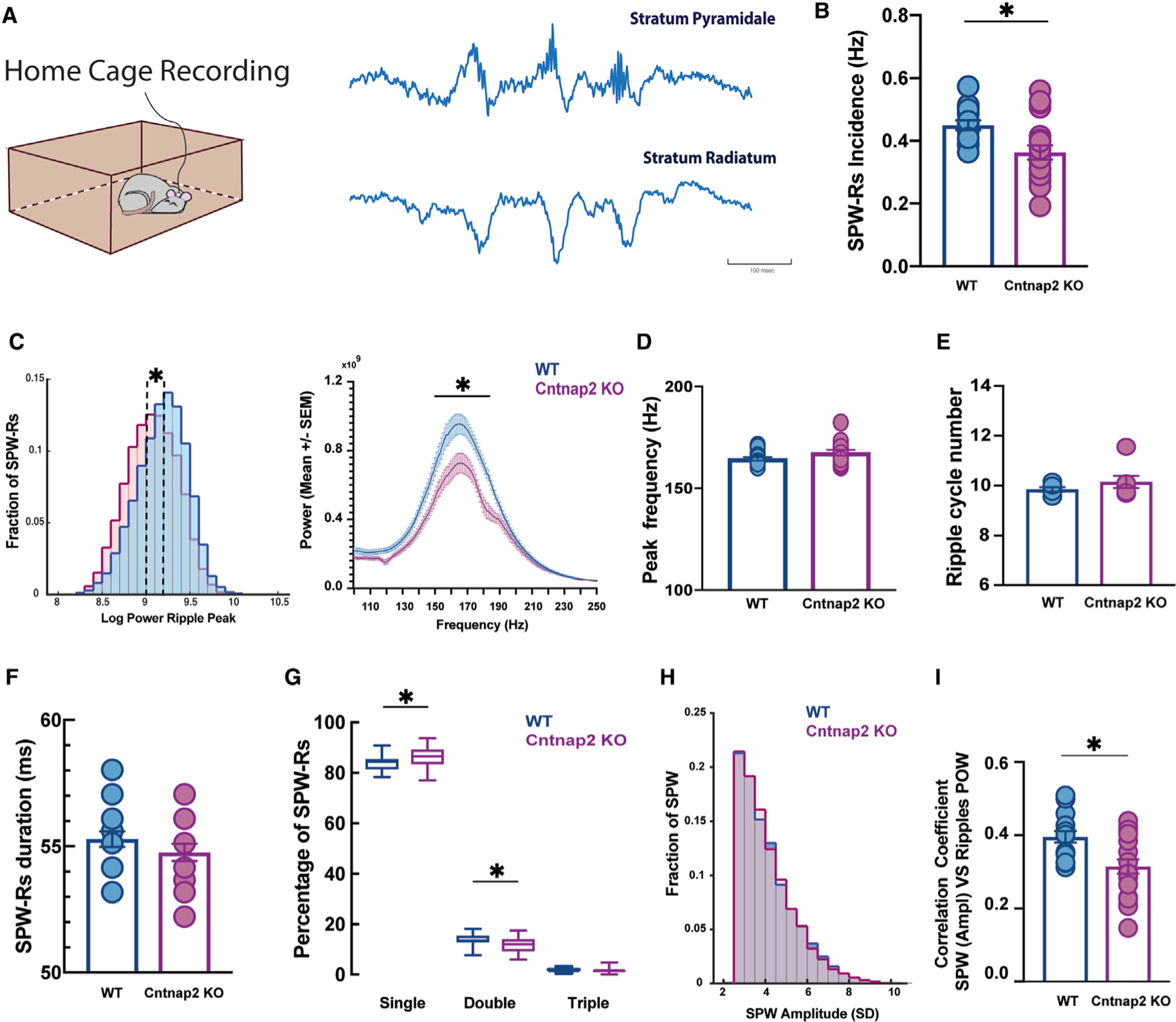
SWR properties recorded in CA1 pyramidal cell layer during immobility in WT and Cntnap2 KO mice (A) Example of LFPs traces containing SWRs in str. pyramidal and str. radiatum during period of immobility recorded in the home cage. (B) SWR incidence (WT, 0.45 ± 0.06 event/s; *Cntnap2* KO, 0.36 ± 0.1 event/s; Mann-Whitney U test, p < 0.01). (C) Left panel: distribution of peak power of SWRs in WT and *Cntnap2* KO (number of events: 13,502 and 14,956, respectively). Dashed lines indicate medians of the distributions, **p < 0.001 rank-sum test. Right panel: average power spectrum for SWRs in WT and *Cntnap2* KO mice per session (WT, 15 sessions recorded in 6 animals; *Cntnap2 KO*, 20 sessions recorded in 7 animals; p < 0.05 in power between 145 Hz and 186 Hz, Holm-Sidak correction for multi t test comparison). (D) SWR peak spectral frequency (calculated with wavelets) (median frequency, WT: 164.5 Hz; *Cntnap2* KO: 167.5 Hz; Mann-Whitney U test, p > 0.05). (E) Number of cycles per ripple event (WT, 9.8 ± 0.07 cycles/ripple; *Cntnap2* KO, 10.1 ± 0.14 cycles/ripple; Mann-Whitney U test, p > 0.05). (F) SWR duration (WT, 55.3 ± 0.3 ms; *Cntnap2* KO, 54.8 ± 0.3 ms; Mann-Whitney U test, p > 0.05). (G) Percentage of SWR occurrence per type. WT singlets, 84% ± 0.01%; *Cntnap2* KO singlets, 86% ± 0.01%; Mann-Whitney U test, p < 0.05. WT doublets, 14% ± 0.001%; *Cntnap2* KO doublets, 12% ± 0.006%; Mann-Whitney U test, p < 0.03. WT triplets, 2% ± 0.001%; *Cntnap2* KO triplets, 2% ± 0.002%; Mann-Whitney U test, p > 0.05. (H) Distribution of the SWP amplitude (median amplitude, WT: 3.80 SD; *Cntnap2* KO: 3.78 SD). (I) Correlation coefficient between SPW amplitude (SD) and average ripple power per session in WT and *Cntnap2* KO. WT, 15 sessions recorded in 6 animals; Cntnap2 KO, 20 sessions recorded in 7 animals; Mann-Whitney U test, p < 0.03. SD, standard deviation. Data are represented as mean ± SEM. Circles represent each recording session. *p < 0.05.

In freely moving animals, phasic inhibition plays a major role in shaping hippocampal SWRs in CA1 (Gan et al., 2017). We therefore investigated SWRs during extended periods of immobility during home-cage recordings (Figure 7A). We found a significant decrease in SWR incidence (Figure 7B) and oscillatory amplitude in *Cntnap2* KO mice (number of SWR events: 13,502 and 14,956 in WT and *Cntnap2* KO, respectively) (Figure 7C). Other features characterizing SWRs, such as duration, number of cycles per event, and frequency, were similar in both conditions (Figures 7D–7F) and consistent with reported benchmarks of SWR activity (Buzsáki, 2015). Bursting events, defined as doublets and triplets, were fewer compared to singlets in both conditions. However, *Cntnap2* KO mice showed an increased number of singlets and a reduced number of doublets compared to age-matched WT controls (Figure 7G).

Given the known positive correlation between the excitatory drive provided by SPWs and the ripple’s oscillatory activity recorded in CA1 (Stark et al., 2014), we next investigated if the difference in ripple power was secondary to a different input drive. Specifically, we compared the magnitude of the sharp waves recorded in str. radiatum in both conditions and found no significant differences (Figure 7H). Furthermore, the correlation coefficient between SPW amplitude and ripple power was statistically significantly smaller for *Cntnap2* KO mice compared to age-matched WT controls (Figure 7I). Together, these results suggest a localized alteration in CA1 pyramidal-to-interneuron interactions during ripple generation in *Cntnap2* KO mice. Given the known epileptic phenotype in *Cntnap2* KO mice with ages older than 8 months (Peñagarikano et al., 2011), we investigated the occurrence of interictal epileptiform discharge (IEDs) and pathological fast ripples (p-ripples) in the CA1 region in younger animals. Using established criteria for the detection of IEDs (Gelinas et al., 2016; Lévesque et al., 2021; see STAR Methods), we did not detect fast ripple events in either WT or *Cntnap2* KO mice during these recording epochs. However, we did identify sporadic IED events in 1 out of 7 *Cntnap2* KO animals (Figure S5).

## DISCUSSION

ASD is a common neurodevelopmental disease involving different brain regions and a spectrum of behavioral deficits (Silverman et al., 2010; Golden et al., 2018). Emerging evidence from mouse models points to a role for interneuron-mediated functional deficits underlying these complex behaviors (Vogt et al., 2015; Paterno et al., 2020; Filice et al., 2020). For example, one recent study using *in vivo* LFP recordings in the medial prefrontal cortex revealed a behavioral deficit correlated with reduced phase-locking to interneuron-mediated delta and theta oscillations (Lazaro et al., 2019). Although *Cntnap2* is expressed in adult hippocampus and embryonic pallial sub-regions where hippocampal interneurons originate, much less is known about these interneurons in ASD. Our study provides functional evidence of reduced PV+ interneuron-mediated perisomatic inhibition onto CA1 pyramidal neurons, decreased theta-nested mid gamma activity, decreased SWR power, and an overall behavioral deficit in spatial memory in the *Cntnap2* KO animal model. This hippocampal-specific microcircuit dysfunction may be amenable to hippocampal-specific therapeutic strategies for ASD

Here, we used *Cntnap*2 KO mice, which is a preclinical animal model with several advantages. First, it is a well-established mouse model that recapitulates key features of ASD (Peñagarikano et al., 2011). Second, previous studies suggested PV-positive interneuron dysfunction in this model (Peñagarikano et al., 2011; Vogt et al., 2018; Lauber et al., 2018). Third, using a rodent model allows us to leverage a substantial body of work linking known *in vivo* electrophysiological properties to underlying hippocampal cellular/network functions (Colgin, 2020). For example, gamma oscillations, SWRs, and theta-gamma cross-frequency coupling correlate to very specific and interneuron-dependent hippocampal network activities; that these findings were present in control animals but altered in *Cntnap2* KO mice both validated our approach and provided a network-level interpretation of our results.

One of our major findings is that hippocampal gamma activity is altered in *Cntnap2* KO mice. Interestingly, these gamma alterations were highly specific in both frequency and the hippocampal sub-layer. In general, gamma oscillations vary in frequency and relation to theta cycles across CA1 hippocampal sub-layers. In str. pyramidale, for example, fast gamma (>100 Hz) is the prominent oscillatory activity recorded with highest power at the trough of the theta cycle, corresponding to the highest probability of pyramidal cell spiking (Lasztóczi and Klausberger, 2014; Schomburg et al., 2014). Str. radiatum primarily receives input from CA3, and slow gamma oscillatory activity has been suggested to link these two regions (Csicsvari et al., 2003; Colgin et al., 2009). Finally, str. LM receives strong glutamatergic input from pyramidal cells located in the medial entorhinal cortex (Suh et al., 2011; Yamamoto et al., 2014), where mid gamma (60–90 Hz) are thought to be generated and transmitted to the hippocampus through the temporo-ammonic pathway (Colgin et al., 2009; Lasztóczi and Klausberger, 2014; Schomburg et al., 2014; Fernández-Ruiz et al., 2017). Using CSD analyses to isolate gamma oscillations from individual hippocampal layers, it was possible to infer input/output and local network activity of the underlying hippocampal circuitry. Hippocampal interneurons appear to be critical to maintain this layer-specific gamma activity as well as hippocampal input/output in general (Colgin, 2016). In particular, PV+ interneurons are primarily responsible for pyramidal cell perisomatic inhibition, whereas SOM-positive cells are primarily involved in controlling distal inhibition onto pyramidal cells (Royer et al., 2012; Fernández-Ruiz et al., 2017). Thus, the balance of inhibition from PV and SOM interneurons is critical in controlling and selecting hippocampal input from upstream regions. By altering PV interneuron density and activity, ASD animals experience a derangement of this balance of inhibition, resulting in abnormal neural oscillations as well as hippocampal input selections, which is not a consequence of a gross difference in animal behavior, given a similar running speed in both conditions.

Consistent with this, acute hippocampal slices from young adult *Cntnap2* KO mice exhibited reduced perisomatic inhibition onto pyramidal cells associated with reduced inhibitory current frequency and amplitude, as well as a reduction in PV-positive interneurons. Given these PV-specific deficits, we expected to find a reduction in mid gamma power and theta-gamma modulation in str. LM during navigation, which was confirmed in Figures 4, 5, and 6. In addition, and perhaps a bit surprisingly, we found increased theta-fast gamma modulation in the pyramidal cell layer that can also be explained, at least in part, by a reduction in PV-positive interneuronal activity, given that PV-positive interneurons have a role in controlling the phase timing of (place) cells in this region. Together, these findings are consistent with reduced perisomatic inhibition in area CA1 leading to an alteration of the proximal and distal dendritic inhibition balance, thereby attenuating entorhinal cortex (EC) input in favor of CA3 input. Of course, reduced input from upstream regions such as entorhinal cortex (Brun et al., 2008) cannot be excluded from consideration as a mechanism here, and future studies will be necessary using simultaneous recordings from both regions during navigation to carefully evaluate this possibility.

The other major finding in our work is a reduction in hippocampal SWR power. SWRs play a critical role in learning and consolidation of hippocampal-dependent memories and in the decision-making process (Joo and Frank, 2018). Depolarization of CA1 pyramidal cells following a recurrent excitatory network of the CA3 area drives the onset of fast activity (ripple event) in pyramidal cell layers. The content of the event is a sequential activity of pyramidal neurons, which is dependent on previous experience (Carr et al., 2012), and PV-positive interneurons play a critical role in ensemble pyramidal cells during SWR events (Stark et al., 2014). In *Cntnap2* KO mice, we found a reduced ripple power, which is considered a measure of pyramidal cell synchrony (Csicsvari et al., 2000; Schomburg et al., 2012), while all the other parameters analyzed were normal, including the degree of depolarization input. These results highlight a local network alteration likely responsible for altering the spike content of ripple events in *Cntnap2* KO animals. The implication of altered ripple activity in ASD is not immediately clear; however, the fact that ASD is associated with a reduction in ripple power suggests that the ‘‘content’’ of the output message of hippocampus to cortex is abnormal in ASD (Siapas and Wilson, 1998).

An additional finding is a reduced discrimination ability in the spatial recognition task in *Cntnap2* KO mice. Spatial object recognition memory is a hippocampal-dependent memory task (Broadbent et al., 2004) in which the CA1 hippocampal region has been shown to be involved in detecting novelty in the spatial environment when one object is moved in a different location (Larkin et al., 2014). Reduced discriminatory ability in these animals further highlights a hippocampal-dependent behavioral phenotype correlated with specific spatial information. Indeed, *Cntnap2* KO mice were able to discriminate when tested in different type of tasks such as the context-dependent task. These animals also showed an autism-like behavioral phenotype previously described as hyperactivity, repetitive behaviors, and altered levels of anxiety. In our study, we did not detect any differences in social interaction using a modified 3-chamber social task, and even if some studies have showed an altered social interaction (Peñagarikano et al., 2011, 2015; Selimbeyoglu et al., 2017), others were not able to detect it (Scott et al., 2019). Additional studies combining different social task protocols to precisely detect which component of the social interaction is impaired in *Cntnap2* KO mice, and at what precise stage of development, are warranted (as suggested in Rein et al., 2020). An additional caveat is that alterations in interneuron-mediated inhibition for *Cntnap2* KO mice were evaluated at a single adult epoch in development, and it is possible that these deficits could lead to additional homeostatic alterations in circuit function (Turrigiano, 2011; Howard et al., 2014; Wu et al., 2020). Further, although a decreased number of PV+ interneurons in the hippocampal region (with a concomitant reduction in synaptic inhibition) is one plausible explanation for the altered oscillatory activity reported in this study, additional explanations are possible. For example, changes in unitary connections between inhibitory neurons and pyramidal cells, or changes in release probability at inhibitory synapses, could contribute to the observed alterations in hippocampal oscillations. In addition, PV+ interneurons include a subpopulation of interneurons such as basket, axoaxonic, and bistratified neurons with different morphological features (Que et al., 2021) and potentially distinct sub-roles in the overall modulation of network dynamics (Forro et al., 2015). As information emerges on the functional roles of distinct subpopulations of PV+ interneurons (and the experimental tools necessary to selectively manipulate these subpopulations), it will be interesting to study these possibilities in further detail.

In summary, our results show that ASD animals exhibited reduced PV+ density (Figure 1) and perisomatic inhibition (Figure 2), resulting in altered hippocampal gamma (Figures 4, 5, and 6) and SWR (Figure 7) activity, which is likely associated with a hippocampal-dependent deficit in spatial memory performance (Figure 3). Collectively, these findings suggest an involvement of the hippocampal memory system in autism phenotypes. Recent clinical studies have also implicated memory impairment as a common finding in patients with ASD (Crane and Goddard, 2008; Loth et al., 2011; Goddard et al., 2014; Robinson et al., 2017; Cooper and Simons, 2019), including deficits in object location recognition memory (Ring et al., 2015). For example, Cooper et al., (2017) investigated the neurophysiological basis of memory in ASD using an fMRI study while subjects were performing an episodic memory task and found a diminished retrieval performance associated with significantly reduced hippocampal connectivity during episodic memory recollection. Our data represent a detailed description of hippocampal-dependent activity and alterations in memory processing associated with a null *Cntnap2* mutation, and further studies focused on hippocampal dependent function are warranted in ASD.

### Limitations of the study

Some possible limitations should be considered when interpreting our results. First, although interpretations discussed here are concordant with a decreased number of soma-targeting hippocampal PV+ interneurons and reduced inhibition onto CA1 pyramidal neurons, functional alterations in interneuron sub-populations such as the dendrite innervating SOM+ interneurons would also influence circuit and behavior phenotypes. Although not the focus of these studies, examination of the involvement of SOM+ as well as other interneuron sub-populations are warranted in these animals. Second, interneuron function is not fixed, and compensatory PV+ interneuron alterations at different developmental ages are possible in *Cntnap2* KO mice. Third, the theta-gamma alteration observed in str. LM during navigation could be interpreted as a local neuronal network alteration or a consequence of an alteration in some downstream hippocampal region such as entorhinal cortex or a combination of both. Future studies to address these latter issues might benefit from a multi-regional recoding approach.

## STAR★METHODS

### RESOURCE AVAILABILITY

#### Lead contact

Further information and requests for resources should be directed to and will be fulfilled by the lead contact, Rosalia Paterno (Rosalia. paterno@ucsf.edu).

#### Materials availability

This study did not generate new unique reagents.

#### Data and code availability

All data reported in this paper will be shared by the lead contact upon request. This paper does not report original code.

Any additional information required to reanalyze the data reported in this paper is available from the lead contact upon request.

### EXPERIMENTAL MODEL AND SUBJECT DETAILS

All experiments were performed on male adult mice (≥2 months old) maintained in a C57B/6 background. Specifically, rodent genotypes *Cntnap2* KO (Jackson laboratory; 017482), C57BL/6J (Jackson laboratory; 000664), and Parvalbumin tdt (+/‒) (Jackson laboratory; 027395) were used for all experiments. All animals were maintained on a 12-hour light/12-hour dark cycle with no food or water restrictions. All procedures involving animals followed guidelines of the National Institutes of Health and were approved by Institutional Animal Care and Use Committee at University of California, San Francisco (#AN181254–02B).

### METHOD DETAILS

#### Behavioral analysis

Behavioral tests were performed in male adult (P90–120) mutant and age-matched control littermates. All tests were performed during the light phase of the light/dark cycle by a trained operator blind to genotype. The order of testing was (1) open field exploration, (2) self-grooming behavior, (3) object congruence task, (4) elevated plus maze (EPM) task, and (5) modified three-chamber social task. An additional cohort of animals performed a spatial object recognition task. These tasks are further described in the following sections.

#### Open field

To assess locomotor activity, we used an open field consisting of a white acrylic glass arena (40 cm x 40 cm) under uniform dim lighting. Individual mice were placed in the center of the arena and their activity was recorded for 25 min. The total distance traveled, and the animal position were automatically calculated using Ethovision software tracking activity (Noldus).

#### Spatial object recognition task

All animals were moved into the testing room for one hour to acclimate under normal lighting conditions and handled for 5 min each day. Animals were habituated to a square testing apparatus (40 cm x 40 cm) with visual cues present on the walls for a 30-min period (habituation phase) (Bevins and Besheer, 2006). Items in the recording room were maintained constant across the experiment and served as distal cues. Each behavioral experiment consisted of 2 different phases – familiarization (F) and test (T) with a rest period between each phase. During familiarization, animals were allowed to explore for a 10-min period two identical novel objects located in 2 different quadrants of the apparatus. Between familiarization and test, animals were placed back in the home cage for 2 h and the arena and objects were cleaned to avoid any olfactory cues. During testing, one object was left in the previous location (unmoved-object) and the other object was placed in a previously empty quadrant (moved-object). The test phase lasted 5 min, during which time the animals explored the new apparatus setting. We restricted our analysis to the first 3 min of the test phase to avoid habituation (Dix and Aggleton, 1999). All experiments were videotaped with a camera placed above and analyzed post-acquisition by an operator blinded to genotype. The videos were processed using Ethovision software 11.5 (Noldus), which sampled video of the task to identify the animal’s body position at 12 frames/sec and the object exploration was manually identified as the animals approached the object within 3 cm with the nose directed to the object and in an active sniffing behavior. To measure recognition memory (Sivakumaran et al., 2018), we calculated discrimination index (DI) from the time of interaction with the moved object (*T*_*mo*_) and the time of interaction with the unmoved object (*T*_*uo*_) as follows:

$$DI=\left(T_{mo}-T_{uo}\right)/\left(T_{mo}+T_{uo}\right)$$

#### Object congruence task

All animals were moved into the testing room for 1 hr to acclimate under normal lighting conditions and handled for 5 min each day. Experiments were carried out inside sound attenuating shells equipped with two 5W lamps. Two different contexts (40 cm x 40 cm) were used. Context A was made of a smooth white acrylic box and Context B had a grid made of green label tape and each context was cleaned with a different type of odorized cleaner. Two sets of objects were built (Object A and Object B) using Lego Megablocks each with a different shape and color/contrast; both set of objects contained an identical odor (20 μL vanilla extract place on the tip of a cotton swab) inserted inside each object. The task consisted of three 10-min trials with a period of home cage resting between each trial. Specifically, during trial 1, animals were exposed to one context containing two identical paired objects (i.e., Context A with Objects A); during trial 2, animals were exposed to another context (never encountered before, i.e., Context B with Objects B) and during test, a context was randomly chosen (Context A or Context B) containing one object A and one Object B. During test, objects were then defined as congruent (object previously shown with the same context i.e., Object A in Context A) or incongruent (object incongruent with the current context, i.e., Object B in Context A). Home cage resting time was 30-min between trial 1 and trial 2 and 2-hr between trial 2 and test. All experiments were videotaped with a camera placed above and analyzed post-acquisition by an operator blinded to genotype. The videos were processed using Ethovision software 11.5 (Noldus), which sampled video of the task to identify the animal’s body position at 12 frames/sec and the object exploration was manually identified as the animals approached the object within 3 cm and the nose directed to the object and in an active sniffing behavior.

#### Elevated plus maze task

The plus maze consisted of 4-arms elevated 50-cm from the floor with 2-closed arms with walls and 2-open arms (with no walls) connected by a junction part. All animals were placed in the junction part of the maze and animal position was recorded for 10 min using a tracking system that automatically calculates time spent in each arm, distance traveled, and number of entries in each arm.

#### Self-grooming behavior

All animals were moved into the testing room for 1 hr to acclimate under normal lighting conditions and handled for 5 min each day. They were then placed individually in a standard mouse cage (13 × 32 × 18 cm) without bedding and recorded for 20 min with a camera placed 1.5 m away on the side of the cage. Videos were then processed post-acquisition by an operator blinded to genotype. Self-grooming was scored for 10-min periods as a measure for grooming in all body parts after 10-min habituation period in the home cage. Number and duration of each episode was reported. If the time between consecutive events was less than 5 s, the events were combined and reported as single event.

#### Modified three-chamber social task

Social interaction was assessed using a modified-three chamber task. The task utilized a Plexiglas box composed of two interconnected chambers with one plastic cup in each chamber and consisted of two 10-min phases: Habituation (H) and Test (T). During Habituation (H), animals were allowed to explore the two chambers containing the two-empty cups for a 10-min period. During Test (T), animals were allowed to explore the two chambers, containing one empty cup in one chamber and one cup with an unfamiliar mouse in the other chamber. All experiments were videotaped with a camera placed above the chambers and analyzed post-acquisition by an operator blinded to genotype. Videos were processed using Ethovision software 11.5 (Noldus), which sampled video of the task to identify the animal’s body position at 12 frames/sec. The time and distance each animal spent in each chamber was calculated and interaction time with the empty or social cup was manually identified as animals approached the cup within 3 cm with nose directed to the object and in an active sniffing behavior.

#### Hippocampal slice preparation

Horizontal hippocampal-entorhinal cortex slices were prepared from *Cntnap2* KO mice and age-matched WT sibling controls. Following deep anesthesia with isoflurane, mice were euthanized by decapitation. The brain was rapidly dissected and immersed in ice-cold oxygenated (95% O_2_/ 5% CO_2_) high-sucrose artificial cerebrospinal solution (sACSF) containing (in mM) 220 sucrose, 3 KCl, 26 NaHCO_3_, 10 dextrose, 2 MgSO_4_, 1.25 NaH_2_PO_4_ and 1 CaCl_2_. 320 mm thickness slices were made using a VT 1000S microtome (Leica) and immediately after transferred to an incubation chamber with artificial cerebrospinal fluid (ACSF) containing (in mM) 124 NaCl, 26 NaHCO_3_, 10 dextrose, 3 KCl, 1 CaCl_2_, 2 MgSO_4_, and 1.25 NaH_2_PO_4_, and maintained at 35°C for 30 min, and thereafter at room temperature for at least 40 min before recording. *Ex vivo* recordings were performed in slices from P90–110 WT and *Cntnap2* KO mice, or P60–90 WT PV-tdt (+/‒) and *Cntnap2* KO PVtdt (+/‒) mice.

#### Patch-clamp recording

Slices were transferred individually to the recording chamber, which was continuously perfused with oxygenated ACSF (2–3 ml/min at 28–30°C). Whole-cell patch-clamp recordings were performed from hippocampal CA1 pyramidal neurons or fluorescent-labeled parvalbumin-positive (PV+) interneurons, using an infrared differential interference contrast (IR-DIC) video-microscopy system with an epifluorescent microscope (Olympus BX50-WI) and water-immersion 40x objective. PV+ interneurons were identified by expression of a red fluorescent protein tdTomato. Patch micropipettes (3–6 MΩ) were pulled from borosilicate glass using a computer-controlled micropipette puller (P-1000, Sutter Instrument) and filled with the appropriate internal solution. To measure inhibitory postsynaptic currents (IPSC), internal solution contained (in mM) 117.5 Cs-Gluconate, 11 CsCl_2_, 1 MgCl_2_, 10 HEPES, 11 EGTA, 2 Na_2_ATP, 0.5 Na_2_GTP and 1.25 QX-314 (285–290 mOsm, pH 7.2). Recordings were performed at a holding potential of 0 mV. To measure excitatory postsynaptic currents (EPSC), internal solution contained (in mM) 135 CsCl_2_, 10 NaCl, 2 MgCl_2_, 10 HEPES, 10 EGTA, 2 Na_2_ATP and 0.2 Na_2_GTP (285–290 mOsm, pH 7.2). Recordings were performed at a holding potential of ‒65 mV. Intrinsic membrane properties were recorded in current-clamp mode with internal solution containing (in mM) 120 K-gluconate, 10 KCl 1 MgCl_2_, 0.025 CaCl_2_, 10 HEPES, 0.2 EGTA, 2 Na_2_ATP, 0.2 Na_2_GTP (285–290 mOsm, pH 7.2). Recordings were performed at a holding potential of ‒65 mV, and electrophysiological properties were measured in response to 250-ms current step injections delivered in 10 pA increments from ‒150 to +150 pA. Data were recorded using a Multiclamp 700B amplifier (Molecular Devices) and monitored with pClamp 10.3 software (Molecular Devices). Whole-cell voltage-clamp data were low-pass filtered at 1 kHz and digitally sampled at 10 kHz using a Digidata 1550A (Molecular Devices). Each IPSC event were manually selected using Mini Analysis 6.0.7 (Synaptosoft Software), and a mean of 100 individual events were analyzed for each cell.

#### Surgery for *in vivo* recordings

Six control and seven *Cntnap2* KO male mice between 90–120 days of age were implanted with chronic silicon probe arrays (Vandecasteele et al., 2012) comprised of four-shanks spaced 400 mm apart with eight electrodes sites in each shank spaced 200 mm apart (A4×8 5mm 200–400-703 CM32; from NeuroNexus Technologies). Before implantation, the back of the shank was covered with DiI (lipophilic fluorescent dye - Thermo Fischer Scientific) to subsequently track electrode locations. Adult mice (3–4 months old; 28–30 g) were anesthetized with Ketamine/Xylazine mixture injected intraperitoneally and maintained with isoflurane 0.8 – 1.2% through a nose cone mounted on a mouse stereotaxic frame (Kopf Instruments, USA). The scalp was reflected with a single incision, disinfected with 3% hydrogen peroxide and the periosteum scraped from the scalp. All the following procedures were conducted under 0.5–5x magnification. Skull screws (Machine Screw #000–120× 1/16’’ Flat Head, Component Supply Company) were placed in the scalp to anchor and support the implant, and two additional screws (Fine Science Tools) were placed in the cerebellum for the reference and ground wires. The entire structure was additionally secured with dental adhesive cement (C&B- Metabond, Parkell). A rectangular craniotomy was then performed on the exposed skull, and the dura was opened. Once the brain parenchyma was exposed, the probe was lowered with a micromanipulator (Kopf Instruments) 2.5 mm beneath the skull surface to target the dorsal hippocampus at the following coordinates from bregma AP: – 2.2 mm and ML: 1.3 ‒1.5 mm. The reference and ground wires were then wrapped around the screws previously placed in the cerebellum area. The exposed cortex and electrodes shanks were covered with Kwik-Sil silicone elastomer. The remaining structure was secured with dental cement (Stoelting Co) to ensure headcap stability overtime. Mice were treated with buprenorphine 0.05 – 0.1 mg/kg and ketofen 5 mg/kg for the following two-three days or until no signs of pain were detected. The animals were given at least one-week recovery time before recording.

#### Electrode localization

To identify electrode localization, both physiologic and histological techniques were used (Bragin et al., 1995; Csicsvari et al., 1999; Montgomery et al., 2008; Vandecasteele et al., 2012). Specifically, mice were deeply anesthetized with a mixture of Ketamine/Xylazine and perfused transcardially with saline followed by 4% paraformaldehyde (PFA; Electron Microscopy Science). Brains were post-fixed with PFA 4% and 50 μm thick coronal sections were cut with a VT 1000S vibratome (Leica Microsystems Inc., Buffalo Grove, IL). Right hemibrains (containing the electrodes) were used for DAPI (PureBlu DAPI Nuclear Staining Dye, BioRad) staining to anatomically identify the labeled-electrodes tracks. Stained sections were then examined with a Nikon microscope. In addition, local field potential (LFP), sharp wave ripple (SWR) events (150–250 Hz) were used to functionally identify electrode location. SWRs were identified in the CA1 pyramidal cell layer and the concomitant sharp wave was used to locate electrodes in the deep layers (Csicsvari et al., 1999). Subsequently, SWR-comodulogram was calculated (Gillespie et al., 2016) and dentate spikes were detected in a sliding-window (Bragin et al., 1995). The final electrode positions were found by using (1) the histological sections to determine the vertical axis of the arrays position, (2) SWRs to determine electrodes in area CA1 and (3) dentate spikes to identify electrodes in dentate gyrus layers.

#### *In vivo* behavioral task and analysis

After the one-week post-operative recovery-time was completed, mice were handled for 3-min/day for the duration of the experiment and allowed to habituate to the recording room and the recording apparatus with home cage recordings for 1–1.5 h/day for 5–7 consecutive days. Afterward, they were exposed to a novel/familiar context paradigm. In detail, we placed mice in different boxes with identical dimensions (40 × 40 cm). In each different box, hereafter referred to as different ‘contexts’ for the remainder of the paper, the base and walls varied by color and material type. In addition, we placed unique spatial cues (printed shapes) on the walls. Thus, a unique context was defined by the color and texture of the walls and base, as well as the spatial cues on different faces of the walls. Using this method, we created three different contexts, which were consistent across mice. In the task, animals were first transferred to the recording room in their home cage and allowed to acclimate to the room for 1h before recording. Following this, animals were recorded for 30-min in the home cage. They then were placed in the first context for 25 min, followed by another 30 min within the home cage, followed by another 25 min in the same context, and finally another 15 min in the home cage. This cycle was repeated on three consecutive days, one for each unique context. Thus, on each day the context was changed such that the animal never encountered the same context on different recording days. Of note, all experiments were run during the light cycle from 1:00 to 7:00 PM PST.

#### Recording and data analysis

Animal position and running speed were tracked and recorded during the task by a digital video camera (NeuroMotive Camera, BlackRock Microsystem) which recognized the mouse head-mount LED and sampled the head position at 50 frames/sec. We validated this automatic tracking algorithm using Ethovision software 11.5 (Noldus), which sampled video of the task to identify the animal’s body position at 25 frames/sec.

Electrophysiological recordings were continuously acquired using a Multichannel Acquisition Cerebus System and a Cereplex mu headstage (16-bit resolution; BlackRock Microsystems, Salt Lake City, UT) at 32 KHz. Raw signal was first visually inspected and electrodes with noise were excluded from further analysis. Using custom-written MATLAB-based software (The MathWorks, MA, USA), LFPs were low pass filtered with a cut-off frequency of 40% of the Nyquist rate (413.6 Hz), and then downsampled to 1034 Hz.

#### Sharp wave ripple detection and analysis

The detection of sharp wave ripples (SWRs) was restricted to ‘immobility periods’ during home cage recording defined as speed less than 1.5 cm/sec, duration more than 20 s and low-theta periods. We found all such immobility periods during the home cage recordings from day 3 to 5, to ensure that mice were habituated to the recording setting and room. The LFP from all electrodes during the immobility periods was then bandpass filtered from 150–250 Hz. The baseline mean and standard deviation of these power values were used to define ripple-candidate events as periods when the smoothed envelope in the filtered data was greater than 3 standard deviations (SD) above baseline for at least 15 ms (Cheng and Frank, 2008). The beginning and end of these events were defined as the period of time in which the power surrounding the candidate ripple events was above one SD; events closer than 40 ms were combined.

Sharp waves (SPWs) were detected within these immobility periods in the channels located in CA1- stratum radiatum. The LFP signal was bandpass filtered at 4–50 Hz and events in the range 20–400 ms exceeding 2.5 SD above baseline were included as candidate SPWs (Fernández-Ruiz et al., 2019). SWRs events were then defined as periods in which both an SPW in CA1- stratum radiatum and a ripple in CA1- stratum pyramidale were found. SWR bursts were defined as periods when more than one event was detected in a 200 ms time window; these bursts were classified as doublets and triplets if there were two or three events, respectively (Yamamoto and Tonegawa, 2017). To detect fast ripples, LFPs from electrodes located in stratum pyramidale during periods of immobility were bandpass filtered from 250–600 Hz. Putative fast ripples were identified as periods in which the smoothed envelope in the filtered data was greater than 5 Standard Deviations (SD) above baseline. Events were required to have at least six cycles of oscillations (i.e., six peaks) with each peak having an amplitude greater than 5 SD of the bandpass LFP filter (Lévesque et al., 2011). To confirm that these fast ripple events were distinct from noise artifact, peak power in frequencies greater than 200 Hz had to be larger than peak power in the frequency range 75–125 Hz for each event (Ewell et al., 2019). Each individual identified fast ripple event was visually inspected and misidentified events were removed from further analysis.

Interictal epileptiform discharge (IEDs) were detected during immobility periods previously selected (see above) in the channel located in CA1-stratum pyramidale. LFP signal was first bandpass filtered at 60–80 Hz and IED events were identified when the smoothed envelope in the filtered data was greater than 5 SD above baseline and the event with an unfiltered envelope was greater than 5 SD above baseline (Gelinas et al., 2016; Lévesque et al., 2021). Using these criteria, detected IEDs exhibited waveform features similar to those shown previously (Buzsáki et al., 1991); representative IED examples are shown in Figure S5.

#### Spectral analysis

Theta epochs were detected automatically using a delta (1–4 Hz) and theta (6–10 Hz) ratio followed by manual adjustment with visual inspection of the LFP and power spectrum. These epochs were restricted to epochs in which the animal was walking/running in the open field. Those epochs were then, convolved with a bank of 100 Morlet wavelet filters (Addison, 2017), linearly spaced from 2 to 100 Hz to generate an instantaneous power and phase. The calculated instantaneous power was then averaged across time to obtain an estimate of the power spectrum for each epoch. To normalize power spectra across epochs, we calculated relative power in which each frequency was expressed as percentage of the total spectrum.

To analyze LFP power as a function of individual theta cycles (‘cycle-by-cycle’ analysis), we first extracted theta phase from the LFP recorded in the pyramidal cell layer. We bandpass filtered the signal between 1–40 Hz and linearly interpolated phase between minima and maxima in each theta cycle using a zero-crossing of theta filtered (6–10 Hz) signal (Belluscio et al., 2012). Theta peaks corresponded to 0° and 360° and theta troughs corresponded to 180° and 540° of theta cycles recorded in pyramidal cell layer. The theta phase of the pyramidal layer was used as reference for all analyses reported in the paper. To calculate gamma power in each theta cycle, we applied 65 Morlet wavelets (as previously described) linearly spaced between 20–150 Hz, and the resulting power amplitude values were z-scored across all the cycles in the theta epochs for each recording session. Theta phase was divided into 16 bins and the z-scored wavelet amplitude was averaged for each bin to obtain the distribution of gamma amplitude by theta phase.

To correct for volume conduction of the LFPs recorded across layers, cycle-by-cycle analysis was also conducted using current source density (CSD) signals. Specifically, CSD signals were calculated as follows,

$$CSD\left(n,t\right)=\frac{\left(LFP\left(n-1,t\right)-2LFP\left(n,t\right)+LFP\left(n+1,t\right)\right)}{\text{distanc}{\text{e}}^{2}}$$

where LFP(n) represents the LFP signal from the electrode of interest; LFP(n-1) and LFP(n+1) refer signals from electrodes above and below the electrode of interest, respectively, and distance refers to the distance between electrodes in millimeters. To test whether high frequency activity varied as a function of the theta cycle, three gamma sub-bands were averaged across each theta phase bin as follow: slow gamma (25–55 Hz), mid gamma (65–90 Hz) and fast gamma (> 100 Hz).

To measure cross-frequency coupling between theta and gamma activity, we calculated a modulation index (MI) (Tort et al., 2010). Briefly, we calculated the relationship between LFP phase in the range 6–12 Hz, and LFP amplitude in 5 Hz non-overlapping windows from 20 to 200 Hz during theta epochs. To ensure that each event had enough length for the low frequency calculation, we restricted the analysis to theta epochs longer than 1.5 s and performed the calculation on the CSD signal previously extracted.

#### Immunohistochemistry, imaging and quantification

Adult (P90–120) WT and *Cntnap2* KO mice were deeply anesthetized with a mixture of Ketamine/Xylazine and perfused transcardially with saline followed by 4% paraformaldehyde (PFA; Electron Microscopy Science). Brains were post-fixed with PFA 4% and 50 mm thick coronal sections were cut with a VT 1000S vibratome (Leica Microsystems Inc., Buffalo Grove, IL). Primary antibody used: anti-parvalbumin (Sigma-Aldrich; P3088; 1:500) and RFP (Rockland; 600–401-379; 1:1000). Secondary antibody used: Alexa 488 and Alexa 594 (Invitrogen; A11001; A11012; 1:1000). Images were acquired at 1024 pixels resolution using a Nikon confocal microscope. Quantification analysis was performed using NIS-Elements (Nikon software) in fluorescent label sections (50 μm) imaged with 4X objective. All cells that expressed the marker were counted in every sixth coronal section (300 μm apart). To quantify the PV-positive density in the hippocampus, 3 sections for the dorsal hippocampus were analyzed for each animal and the value averaged to calculate a mean cell density (cell/mm^2^).

#### Graphical abstract

The graphical abstract was prepared using the BioRender software (BioRender.com).

### QUANTIFICATION AND STATISTICAL ANALYSIS

All analyses were performed using MATLAB (MathWorks) and PRISM (GraphPad). We compared effects across session for each of the two animal conditions (wild-type versus KO). Based upon normality, we used parametric or non-parametric tests accordingly as specified in each analysis. For multi-comparison correction, post hoc testing was done using the Tukey or Sidak correction, as indicated in each analysis. All plots with error bars are reported as mean ± SEMs.

## Supplementary Material

1

2

## Figures and Tables

**Table:** KEY RESOURCES TABLE

REAGENT or RESOURCE	SOURCE	IDENTIFIER
Antibodies		

Mouse anti-PV	Millipore-Sigma	Cat#P3088; RRID: AB_477329
Rabbit anti-RFP	Rockland	Cat#600–401-379; RRID: AB_2209751
Alexa Fluor488 goat anti-mouse IgG	Thermo Fisher	Cat#A-11001; RRID: AB_2534069
Alexa Fluor594 goat anti-rabbit IgG	Thermo Fisher	Cat#A-11012; RRID: AB_141359

Chemicals, peptides, and recombinant proteins		

DAPI	BioRad	Cat#1351303
Dil	Thermo Fisher	Cat#D282
Paraformaldehyde Aqueous Solution 16%	Electronic Microscopy Sciences	Cat#: RT15710-S
Sucrose	Fisher Scientific	Cat#S3–500
Potassium Chloride	Millipore Sigma	Cat#PX1405
Calcium Chloride Dihydrate	Fisher Scientific	Cat#BP510–100
Magnesium Sulfate Heptahydrate	Fisher Scientific	Cat#BP213–1
Dextrose	Fisher Scientific	Cat#D15–500
Sodium Bicarbonate	Fisher Scientific	Cat#BP328–500
Sodium Phosphate Monobasic Monohydrate	Fisher Scientific	Cat#BP330–500
Sodium Chloride	Fisher Scientific	Cat#BP358–212
HEPES	Fisher Scientific	Cat#AC172572500
EGTA	Millipore Sigma	Cat#324626
QX-314	Millipore Sigma	Cat#552233
Magnesium Chloride	Fisher Scientific	Cat#BP214–500
Cesium chloride	Millipore Sigma	Cat#C3011
Adenosine 5′-triphosphate disodium salt hydrate	Millipore Sigma	Cat#A26209
Guanosine 5′-triphosphate sodium salt hydrate	Millipore Sigma	Cat#G8877

Deposited data		

Raw and analyzed data	This Paper	Available upon request
MATLAB Code	This Paper	Available upon request

Experimental models: Organisms/strains		

Mouse: C57BL/6J	Jackson laboratory	JAX: 000664
Mouse: *Cntnap2* knock-out	Jackson laboratory	JAX: 017482
Mouse: Pvalb-tdTomato	Jackson laboratory	JAX: 027395

Software and algorithms		

Adobe Illustrator CS6	Adobe Systems	https://www.adobe.com/products/illustrator.html
MATLAB 2017b	MathWorks	https://www.mathworks.com; RRID:SCR_001622
EthoVision XT	Noldus	https://www.noldus.com/animal-behavior-research/
Mini Analysis 6.0.7	Synaptosoft	http://www.synaptosoft.com/MiniAnalysis/
PRISM	Graphpad	https://www.graphpad.com/
NIS-Elements	Nikon Software	https://www.microscope.healthcare.nikon.com/en_EU/products/software/nis-elements
BioRender	BioRender.com	N/A

Other		

Stereotaxic frame	Kopf Instruments	N/A
Vibratome	Leica Biosystems Inc.	Cat#VT1000S
Multichannel Acquisition Cerebus System	BlackRock Microsystems	N/A
Silicon probes: 32-channel; 200 mm inter-spacing; 703 mm2 electrode area	NeuroNexus Technologies	CM32: A4×8 5mm 200–400-703

## Highlights

- Reduced number of PV-positive interneurons in hippocampal area CA1 of *Cntnap2* KO mice
- Reduced frequency and amplitude of IPSCs onto CA1 pyramidal neurons of *Cntnap2* KO mice
- Hippocampal alteration in gamma oscillations and sharp wave ripples in *Cntnap2* KO mice
- Impaired spatial discrimination ability in *Cntnap2* KO mice

## References

[R1] Abrahams BS, Arking DE, Campbell DB, Mefford HC, Morrow EM, Weiss LA, Menashe I, Wadkins T, Banerjee-Basu S, and Packer A (2013). SFARI Gene 2.0: a community-driven knowledgebase for the autism spectrum disorders (ASDs). Mol. Autism 4, 36. 24090431 10.1186/2040-2392-4-36 PMC3851189

[R2] Addison PS (2017). The Illustrated Wavelet Transform Handbook: Introductory Theory and Applications in Science (Engineering, Medicine and Finance).

[R3] Ariza J, Rogers H, Hashemi E, Noctor SC, and Martínez-Cerdeño V (2018). The Number of Chandelier and Basket Cells Are Differentially Decreased in Prefrontal Cortex in Autism. Cereb. Cortex 28, 411–420. 28122807 10.1093/cercor/bhw349 PMC6676950

[R4] Arking DE, Cutler DJ, Brune CW, Teslovich TM, West K, Ikeda M, Rea A, Guy M, Lin S, Cook EH, and Chakravarti A (2008). A common genetic variant in the neurexin superfamily member CNTNAP2 increases familial risk of autism. Am. J. Hum. Genet 82, 160–164. 18179894 10.1016/j.ajhg.2007.09.015 PMC2253968

[R5] Baio J, Wiggins L, Christensen DL, Maenner MJ, Daniels J, Warren Z, Kurzius-Spencer M, Zahorodny W, Rosenberg CR, White T, (2018). Prevalence of Autism Spectrum Disorder Among Children Aged 8 Years ‒ Autism and Developmental Disabilities Monitoring Network, 11 Sites, United States, 2014. Surveillance Summaries 67, 1–23. 10.15585/mmwr.ss6706a1 PMC5919599 29701730

[R6] Belluscio MA, Mizuseki K, Schmidt R, Kempter R, and Buzsáki G (2012). Cross-frequency phase-phase coupling between θ and γ oscillations in the hippocampus. J. Neurosci 32, 423–435. 22238079 10.1523/JNEUROSCI.4122-11.2012 PMC3293373

[R7] Bevins RA, and Besheer J (2006). Object recognition in rats and mice: a one-trial non-matching-to-sample learning task to study ‘recognition memory’. Nat. Protoc 1, 1306–1311. 17406415 10.1038/nprot.2006.205

[R8] Bezaire MJ, and Soltesz I (2013). Quantitative assessment of CA1 local circuits: knowledge base for interneuron-pyramidal cell connectivity. Hippocampus 23, 751–785. 23674373 10.1002/hipo.22141 PMC3775914

[R9] Blatt GJ, Fitzgerald CM, Guptill JT, Booker AB, Kemper TL, and Bauman ML (2001). Density and Distribution of Hippocampal Neurotransmitter Receptors in Autism: An Autoradiographic Study. J. Autism Dev. Disord 7, 537–543. 10.1023/a:1013238809666 11814263

[R10] Bragin A, Jandó G, Nádasdy Z, van Landeghem M, and Buzsáki G (1995). Dentate EEG spikes and associated interneuronal population bursts in the hippocampal hilar region of the rat. J. Neurophysiol 73, 1691–1705. 7643175 10.1152/jn.1995.73.4.1691

[R11] Broadbent NJ, Squire LR, and Clark RE (2004). Spatial memory, recognition memory, and the hippocampus. Proc. Natl. Acad. Sci. USA 101, 14515– 14520. 15452348 10.1073/pnas.0406344101 PMC521976

[R12] Brun VH, Leutgeb S, Wu H-Q, Schwarcz R, Witter MP, Moser EI, and Moser M-B (2008). Impaired spatial representation in CA1 after lesion of direct input from entorhinal cortex. Neuron 57, 290–302. 18215625 10.1016/j.neuron.2007.11.034

[R13] Burgess N (2008). Spatial cognition and the brain. Ann. N Y Acad. Sci 1124, 77–97. 18400925 10.1196/annals.1440.002

[R14] Buzsáki G (2002). Theta oscillations in the hippocampus. Neuron 33, 325–340. 11832222 10.1016/s0896-6273(02)00586-x

[R15] Buzsáki G (2015). Hippocampal sharp wave-ripple: A cognitive biomarker for episodic memory and planning. Hippocampus 25, 1073–1188. 26135716 10.1002/hipo.22488 PMC4648295

[R16] Buzsáki G, Hsu M, Slamka C, Gage FH, and Horváth Z (1991). Emergence and propagation of interictal spikes in the subcortically denervated hippocampus. Hippocampus 1, 163–180. 1669291 10.1002/hipo.450010205

[R17] Cardin JA, Carlén M, Meletis K, Knoblich U, Zhang F, Deisseroth K, Tsai L-H, and Moore CI (2009). Driving fast-spiking cells induces gamma rhythm and controls sensory responses. Nature 459, 663–667. 19396156 10.1038/nature08002 PMC3655711

[R18] Carr MF, Karlsson MP, and Frank LM (2012). Transient slow gamma synchrony underlies hippocampal memory replay. Neuron 75, 700–713. 22920260 10.1016/j.neuron.2012.06.014 PMC3428599

[R19] Chao H-T, Chen H, Samaco RC, Xue M, Chahrour M, Yoo J, Neul JL, Gong S, Lu H-C, Heintz N, (2010). Dysfunction in GABA signalling mediates autism-like stereotypies and Rett syndrome phenotypes. Nature 468, 263–269. 21068835 10.1038/nature09582 PMC3057962

[R20] Cheng S, and Frank LM (2008). New experiences enhance coordinated neural activity in the hippocampus. Neuron 57, 303–313. 18215626 10.1016/j.neuron.2007.11.035 PMC2244590

[R21] Cobb SR, Buhl EH, Halasy K, Paulsen O, and Somogyi P (1995). Synchronization of neuronal activity in hippocampus by individual GABAergic interneurons. Nature 378, 75–78. 7477292 10.1038/378075a0

[R22] Colgin LL (2016). Rhythms of the hippocampal network. Nat. Rev. Neurosci 17, 239–249. 26961163 10.1038/nrn.2016.21 PMC4890574

[R23] Colgin LL (2020). Five Decades of Hippocampal Place Cells and EEG Rhythms in Behaving Rats. J. Neurosci 40, 54–60. 31451578 10.1523/JNEUROSCI.0741-19.2019 PMC6939480

[R24] Colgin LL, Denninger T, Fyhn M, Hafting T, Bonnevie T, Jensen O, Moser M-B, and Moser EI (2009). Frequency of gamma oscillations routes flow of information in the hippocampus. Nature 462, 353–357. 19924214 10.1038/nature08573

[R25] Cooper RA, and Simons JS (2019). Exploring the neurocognitive basis of episodic recollection in autism. Psychon. Bull. Rev 26, 163–181. 29987766 10.3758/s13423-018-1504-z PMC6424931

[R26] Cooper RA, Richter FR, Bays PM, Plaisted-Grant KC, Baron-Cohen S, and Simons JS (2017). Reduced Hippocampal Functional Connectivity During Episodic Memory Retrieval in Autism. Cereb. Cortex 27, 888–902. 28057726 10.1093/cercor/bhw417 PMC5390398

[R27] Crane L, and Goddard L (2008). Episodic and semantic autobiographical memory in adults with autism spectrum disorders. J. Autism Dev. Disord 38, 498–506. 17668308 10.1007/s10803-007-0420-2

[R28] Csicsvari J, Hirase H, Czurkó A, Mamiya A, and Buzsáki G (1999). Oscillatory coupling of hippocampal pyramidal cells and interneurons in the behaving Rat. J. Neurosci 19, 274–287. 9870957 10.1523/JNEUROSCI.19-01-00274.1999 PMC6782375

[R29] Csicsvari J, Hirase H, Mamiya A, and Buzsáki G (2000). Ensemble patterns of hippocampal CA3-CA1 neurons during sharp wave-associated population events. Neuron 28, 585–594. 11144366 10.1016/s0896-6273(00)00135-5

[R30] Csicsvari J, Jamieson B, Wise KD, and Buzsáki G (2003). Mechanisms of gamma oscillations in the hippocampus of the behaving rat. Neuron 37, 311–322. 12546825 10.1016/s0896-6273(02)01169-8

[R31] Dix SL, and Aggleton JP (1999). Extending the spontaneous preference test of recognition: evidence of object-location and object-context recognition. Behav. Brain Res 99, 191–200. 10512585 10.1016/s0166-4328(98)00079-5

[R32] Ewell LA, Fischer KB, Leibold C, Leutgeb S, and Leutgeb JK (2019). The impact of pathological high-frequency oscillations on hippocampal network activity in rats with chronic epilepsy. eLife 8, e42148. 30794155 10.7554/eLife.42148 PMC6386518

[R33] Fatemi SH, Reutiman TJ, Folsom TD, Rooney RJ, Patel DH, and Thuras PD (2010). mRNA and protein levels for GABAAalpha4, α5, β1 and GABABR1 receptors are altered in brains from subjects with autism. J. Autism Dev. Disord 40, 743–750. 20066485 10.1007/s10803-009-0924-z PMC2865581

[R34] Fernández-Ruiz A, Oliva A, Nagy GA, Maurer AP, Berényi A, and Buzsáki G (2017). Entorhinal-CA3 Dual-Input Control of Spike Timing in the Hippocampus by Theta-Gamma Coupling. Neuron 93, 1213–1226.e5. 28279355 10.1016/j.neuron.2017.02.017 PMC5373668

[R35] Fernández-Ruiz A, Oliva A, Fermino de Oliveira E, Rocha-Almeida F, Tingley D, and Buzsáki G (2019). Long-duration hippocampal sharp wave ripples improve memory. Science 364, 1082–1086. 31197012 10.1126/science.aax0758 PMC6693581

[R36] Filice F (2016). Reduction in parvalbumin expression not loss of the parvalbumin-expressing GABA interneuron subpopulation in genetic parvalbumin and shank mouse models of autism. Molecular Brain 9, 10. 26819149 10.1186/s13041-016-0192-8 PMC4729132

[R37] Filice F, Janickova L, Henzi T, Bilella A, and Schwaller B (2020). The Parvalbumin Hypothesis of Autism Spectrum Disorder. Front. Cell. Neurosci 14, 577525. 33390904 10.3389/fncel.2020.577525 PMC7775315

[R38] Forro T, Valenti O, Lasztoczi B, and Klausberger T (2015). Temporal organization of GABAergic interneurons in the intermediate CA1 hippocampus during network oscillations. Cereb. Cortex 25, 1228–1240. 24275828 10.1093/cercor/bht316

[R39] Freund TF, and Katona I (2007). Perisomatic inhibition. Neuron 56, 33–42. 17920013 10.1016/j.neuron.2007.09.012

[R40] Gan J, Weng SM, Pernía-Andrade AJ, Csicsvari J, and Jonas P (2017). Phase-Locked Inhibition, but Not Excitation, Underlies Hippocampal Ripple Oscillations in Awake Mice In Vivo. Neuron 93, 308–314. 28041883 10.1016/j.neuron.2016.12.018 PMC5263253

[R41] Gelinas JN, Khodagholy D, Thesen T, Devinsky O, and Buzsáki G (2016). Interictal epileptiform discharges induce hippocampal-cortical coupling in temporal lobe epilepsy. Nat. Med 22, 641–648. 27111281 10.1038/nm.4084 PMC4899094

[R42] Gelman DM, and Marín O (2010). Generation of interneuron diversity in the mouse cerebral cortex. Eur. J. Neurosci 31, 2136–2141. 20529125 10.1111/j.1460-9568.2010.07267.x

[R43] Gillespie AK, Jones EA, Lin Y-H, Karlsson MP, Kay K, Yoon SY, Tong LM, Nova P, Carr JS, Frank LM, and Huang Y (2016). Apolipoprotein E4 Causes Age-Dependent Disruption of Slow Gamma Oscillations during Hippocampal Sharp-Wave Ripples. Neuron 90, 740–751. 27161522 10.1016/j.neuron.2016.04.009 PMC5097044

[R44] Goddard L, Dritschel B, Robinson S, and Howlin P (2014). Development of autobiographical memory in children with autism spectrum disorders: deficits, gains, and predictors of performance. Dev. Psychopathol 26, 215–228. 24284059 10.1017/S0954579413000904

[R45] Gogolla N, Leblanc JJ, Quast KB, Südhof TC, Fagiolini M, and Hensch TK (2009). Common circuit defect of excitatory-inhibitory balance in mouse models of autism. J. Neurodev. Disord 1, 172–181. 20664807 10.1007/s11689-009-9023-x PMC2906812

[R46] Golden CE, Buxbaum JD, and De Rubeis S (2018). Disrupted circuits in mouse models of autism spectrum disorder and intellectual disability. Curr. Opin. Neurobiol 48, 106–112. 29222989 10.1016/j.conb.2017.11.006 PMC5825272

[R47] Gordon A, Salomon D, Barak N, Pen Y, Tsoory M, Kimchi T, and Peles E (2016). Expression of Cntnap2 (Caspr2) in multiple levels of sensory systems. Mol. Cell. Neurosci 70, 42–53. 26647347 10.1016/j.mcn.2015.11.012

[R48] Guptill JT, Booker AB, Gibbs TT, Kemper TL, Bauman ML, and Blatt GJ (2007). [3H]-flunitrazepam-labeled benzodiazepine binding sites in the hippocampal formation in autism: a multiple concentration autoradiographic study. J. Autism Dev. Disord 37, 911–920. 17019626 10.1007/s10803-006-0226-7

[R49] Harada M, Taki MM, Nose A, Kubo H, Mori K, Nishitani H, and Matsuda T (2011). Non-invasive evaluation of the GABAergic/glutamatergic system in autistic patients observed by MEGA-editing proton MR spectroscopy using a clinical 3 tesla instrument. J. Autism Dev. Disord 41, 447–454. 20652388 10.1007/s10803-010-1065-0

[R50] Hashemi E, Ariza J, Rogers H, Noctor SC, and Martínez-Cerdeño V (2018). The Number of Parvalbumin-Expressing Interneurons Is Decreased in the Prefrontal Cortex in Autism. Cereb. Cortex 28, 690. 28334402 10.1093/cercor/bhx063 PMC6790539

[R51] Hasselmo ME, Bodelón C, and Wyble BP (2002). A proposed function for hippocampal theta rhythm: separate phases of encoding and retrieval enhance reversal of prior learning. Neural Comput 14, 793–817. 11936962 10.1162/089976602317318965

[R52] Howard MA, Rubenstein JLR, and Baraban SC (2014). Bidirectional homeostatic plasticity induced by interneuron cell death and transplantation in vivo. Proc. Natl. Acad. Sci. USA 111, 492–497. 24344303 10.1073/pnas.1307784111 PMC3890856

[R53] Hyman SL, Levy SE, and Myers SM; COUNCIL ON CHILDREN WITH DISABILITIES, SECTION ON DEVELOPMENTAL AND BEHAVIORAL PEDIATRICS (2020). Identification, Evaluation, and Management of Children With Autism Spectrum Disorder. Pediatrics 145, e20193447. 31843864 10.1542/peds.2019-3447

[R54] Iakoucheva LM, Muotri AR, and Sebat J (2019). Getting to the Cores of Autism. Cell 178, 1287–1298. 31491383 10.1016/j.cell.2019.07.037 PMC7039308

[R55] Ito-Ishida A, Ure K, Chen H, Swann JW, and Zoghbi HY (2015). Loss of MeCP2 in Parvalbumin-and Somatostatin-Expressing Neurons in Mice Leads to Distinct Rett Syndrome-like Phenotypes. Neuron 88, 651–658. 26590342 10.1016/j.neuron.2015.10.029 PMC4656196

[R56] Joo HR, and Frank LM (2018). The hippocampal sharp wave-ripple in memory retrieval for immediate use and consolidation. Nat. Rev. Neurosci 19, 744–757. 30356103 10.1038/s41583-018-0077-1 PMC6794196

[R57] Jurgensen S, and Castillo PE (2015). Selective Dysregulation of Hippocampal Inhibition in the Mouse Lacking Autism Candidate Gene CNTNAP2. J. Neurosci 35, 14681–14687. 26511255 10.1523/JNEUROSCI.1666-15.2015 PMC4623232

[R58] Kalueff AV, Stewart AM, Song C, Berridge KC, Graybiel AM, and Fentress JC (2016). Neurobiology of rodent self-grooming and its value for translational neuroscience. Nat. Rev. Neurosci 17, 45–59. 26675822 10.1038/nrn.2015.8 PMC4840777

[R59] Kim YS, Leventhal BL, Koh Y-J, Fombonne E, Laska E, Lim E-C, Cheon K-A, Kim S-J, Kim Y-K, Lee H, (2011). Prevalence of autism spectrum disorders in a total population sample. Am. J. Psychiatry 168, 904–912. 21558103 10.1176/appi.ajp.2011.10101532

[R60] Klausberger T, Magill PJ, Márton LF, Roberts JDB, Cobden PM, Buzsáki G, and Somogyi P (2003). Brain-state- and cell-type-specific firing of hippocampal interneurons in vivo. Nature 421, 844–848. 12594513 10.1038/nature01374

[R61] Kosaka T, Katsumaru H, Hama K, Wu J-Y, and Heizmann CW (1987). GABAergic neurons containing the Ca2+-binding protein parvalbumin in the rat hippocampus and dentate gyrus. Brain Res 419, 119–130. 3315112 10.1016/0006-8993(87)90575-0

[R62] Larkin MC, Lykken C, Tye LD, Wickelgren JG, and Frank LM (2014). Hippocampal output area CA1 broadcasts a generalized novelty signal during an object-place recognition task. Hippocampus 24, 773–783. 24596296 10.1002/hipo.22268 PMC4065199

[R63] Lasztóczi B, and Klausberger T (2014). Layer-specific GABAergic control of distinct gamma oscillations in the CA1 hippocampus. Neuron 81, 1126–1139. 24607232 10.1016/j.neuron.2014.01.021

[R64] Lauber E, Filice F, and Schwaller B (2016). Prenatal Valproate Exposure Differentially Affects Parvalbumin-Expressing Neurons and Related Circuits in the Cortex and Striatum of Mice. Front. Mol. Neurosci 9, 150. 28066177 10.3389/fnmol.2016.00150 PMC5174119

[R65] Lauber E, Filice F, and Schwaller B (2018). Dysregulation of Parvalbumin Expression in the Cntnap2−/− Mouse Model of Autism Spectrum Disorder. Front. Mol. Neurosci 11, 262. 30116174 10.3389/fnmol.2018.00262 PMC6082962

[R66] Lawrence YA, Kemper TL, Bauman ML, and Blatt GJ (2010). Parvalbumin-, calbindin-, and calretinin-immunoreactive hippocampal interneuron density in autism. Acta Neurol. Scand 121, 99–108. 19719810 10.1111/j.1600-0404.2009.01234.x

[R67] Lazaro MT, Taxidis J, Shuman T, Bachmutsky I, Ikrar T, Santos R, Marcello GM, Mylavarapu A, Chandra S, Foreman A, (2019). Reduced Prefrontal Synaptic Connectivity and Disturbed Oscillatory Population Dynamics in the CNTNAP2 Model of Autism. Cell Rep 27, 2567–2578.e6. 31141683 10.1016/j.celrep.2019.05.006 PMC6553483

[R68] Lee E, Lee J, and Kim E (2017). Excitation/Inhibition Imbalance in Animal Models of Autism Spectrum Disorders. Biol. Psychiatry 81, 838–847. 27450033 10.1016/j.biopsych.2016.05.011

[R69] Lévesque M, Bortel A, Gotman J, and Avoli M (2011). High-frequency (80–500 Hz) oscillations and epileptogenesis in temporal lobe epilepsy. Neurobiol. Dis 42, 231–241. 21238589 10.1016/j.nbd.2011.01.007 PMC4873283

[R70] Lévesque M, Macey-Dare ADB, Wang S, and Avoli M (2021). Evolution of interictal spiking during the latent period in a mouse model of mesial temporal lobe epilepsy. Curr. Res. Neurobiol 2, 100008. 10.1016/j.crneur.2021.100008 PMC9559106 36246508

[R71] Li X-G, Somogyi P, Tepper JM, and Buzsáki G (1992). Axonal and dendritic arborization of an intracellularly labeled chandelier cell in the CA1 region of rat hippocampus. Exp. Brain Res 90, 519–525. 1385200 10.1007/BF00230934

[R72] Loth E, Gómez JC, and Happé F (2011). Do high-functioning people with autism spectrum disorder spontaneously use event knowledge to selectively attend to and remember context-relevant aspects in scenes? J. Autism Dev. Disord 41, 945–961. 21042873 10.1007/s10803-010-1124-6

[R73] Miles R, Tóth K, Gulyás AI, Hájos N, and Freund TF (1996). Differences between somatic and dendritic inhibition in the hippocampus. Neuron 16, 815–823. 8607999 10.1016/s0896-6273(00)80101-4

[R74] Montgomery SM, Sirota A, and Buzsáki G (2008). Theta and gamma coordination of hippocampal networks during waking and rapid eye movement sleep. J. Neurosci 28, 6731–6741. 18579747 10.1523/JNEUROSCI.1227-08.2008 PMC2596978

[R75] O’Keefe J, and Nadel L (1978). The hippocampus as a cognitive map (Clarendon Press; Oxford University Press).

[R76] Paterno R, Casalia M, and Baraban SC (2020). Interneuron deficits in neurodevelopmental disorders: Implications for disease pathology and interneuron-based therapies. Eur. J. Paediatr. Neurol 24, 81–88. 31870698 10.1016/j.ejpn.2019.12.015 PMC7152321

[R77] Peñagarikano O, Abrahams BS, Herman EI, Winden KD, Gdalyahu A, Dong H, Sonnenblick LI, Gruver R, Almajano J, Bragin A, (2011). Absence of CNTNAP2 leads to epilepsy, neuronal migration abnormalities, and core autism-related deficits. Cell 147, 235–246. 21962519 10.1016/j.cell.2011.08.040 PMC3390029

[R78] Peñagarikano O, Lázaro MT, Lu X-H, Gordon A, Dong H, Lam HA, Peles E, Maidment NT, Murphy NP, Yang XW, (2015). Exogenous and evoked oxytocin restores social behavior in the Cntnap2 mouse model of autism. Sci. Transl. Med 7, 271ra8. 10.1126/scitranslmed.3010257 PMC4498455 25609168

[R79] Que L, Lukacsovich D, Luo W, and Földy C (2021). Transcriptional and morphological profiling of parvalbumin interneuron subpopulations in the mouse hippocampus. Nat. Commun 12, 108. 33398060 10.1038/s41467-020-20328-4 PMC7782706

[R80] Rácz A, Ponomarenko AA, Fuchs EC, and Monyer H (2009). Augmented hippocampal ripple oscillations in mice with reduced fast excitation onto parvalbumin-positive cells. J. Neurosci 29, 2563–2568. 19244531 10.1523/JNEUROSCI.5036-08.2009 PMC6666231

[R81] Rein B, Ma K, and Yan Z (2020). A standardized social preference protocol for measuring social deficits in mouse models of autism. Nat. Protoc 15, 3464–3477. 32895524 10.1038/s41596-020-0382-9 PMC8103520

[R82] Ring M, Gaigg SB, and Bowler DM (2015). Object-location memory in adults with autism spectrum disorder. Autism Res 8, 609–619. 25820615 10.1002/aur.1478

[R83] Robinson S, Howlin P, and Russell A (2017). Personality traits, autobiographical memory and knowledge of self and others: A comparative study in young people with autism spectrum disorder. Autism 21, 357–367. 27197697 10.1177/1362361316645429

[R84] Royer S, Zemelman BV, Losonczy A, Kim J, Chance F, Magee JC, and Buzsáki G (2012). Control of timing, rate and bursts of hippocampal place cells by dendritic and somatic inhibition. Nat. Neurosci 15, 769–775. 22446878 10.1038/nn.3077 PMC4919905

[R85] Rubenstein JLR, and Merzenich MM (2003). Model of autism: increased ratio of excitation/inhibition in key neural systems. Genes Brain Behav 2, 255–267. 14606691 10.1034/j.1601-183x.2003.00037.x PMC6748642

[R86] Schendel DE, and Thorsteinsson E (2018). Cumulative Incidence of Autism Into Adulthood for Birth Cohorts in Denmark, 1980–2012. JAMA 320, 1811–1813. 30398592 10.1001/jama.2018.11328 PMC6248103

[R87] Schomburg EW, Anastassiou CA, Buzsáki G, and Koch C (2012). The spiking component of oscillatory extracellular potentials in the rat hippocampus. J. Neurosci 32, 11798–11811. 22915121 10.1523/JNEUROSCI.0656-12.2012 PMC3459239

[R88] Schomburg EW, Fernández-Ruiz A, Mizuseki K, Berényi A, Anastassiou CA, Koch C, and Buzsáki G (2014). Theta phase segregation of input-specific gamma patterns in entorhinal-hippocampal networks. Neuron 84, 470–485. 25263753 10.1016/j.neuron.2014.08.051 PMC4253689

[R89] Scott R, Sánchez-Aguilera A, van Elst K, Lim L, Dehorter N, Bae SE, Bartolini G, Peles E, Kas MJH, Bruining H, and Marín O (2019). Loss of Cntnap2 Causes Axonal Excitability Deficits, Developmental Delay in Cortical Myelination, and Abnormal Stereotyped Motor Behavior. Cereb. Cortex 29, 586–597. 29300891 10.1093/cercor/bhx341

[R90] Seibenhener ML, and Wooten MC (2015). Use of the Open Field Maze to measure locomotor and anxiety-like behavior in mice. J. Vis. Exp 96, e52434. 10.3791/52434 PMC4354627 25742564

[R91] Selimbeyoglu A, Kim CK, Inoue M, Lee SY, Hong ASO, Kauvar I, Ramakrishnan C, Fenno LE, Davidson TJ, Wright M, and Deisseroth K (2017). Modulation of prefrontal cortex excitation/inhibition balance rescues social behavior in CNTNAP2-deficient mice. Sci. Transl. Med 9, eaah6733. 10.1126/scitranslmed.aah6733 PMC5723386 28768803

[R92] Siapas AG, and Wilson MA (1998). Coordinated interactions between hippocampal ripples and cortical spindles during slow-wave sleep. Neuron 21, 1123–1128. 9856467 10.1016/s0896-6273(00)80629-7

[R93] Silverman JL, Yang M, Lord C, and Crawley JN (2010). Behavioural phenotyping assays for mouse models of autism. Nat. Rev. Neurosci 11, 490–502. 20559336 10.1038/nrn2851 PMC3087436

[R94] Sivakumaran MH, Mackenzie AK, Callan IR, Ainge JA, and O’Connor AR (2018). The Discrimination Ratio derived from Novel Object Recognition tasks as a Measure of Recognition Memory Sensitivity, not Bias. Sci. Rep 8, 11579. 30069031 10.1038/s41598-018-30030-7 PMC6070491

[R95] Sohal VS, and Rubenstein JLR (2019). Excitation-inhibition balance as a framework for investigating mechanisms in neuropsychiatric disorders. Mol. Psychiatry 24, 1248–1257. 31089192 10.1038/s41380-019-0426-0 PMC6742424

[R96] Sohal VS, Zhang F, Yizhar O, and Deisseroth K (2009). Parvalbumin neurons and gamma rhythms enhance cortical circuit performance. Nature 459, 698–702. 19396159 10.1038/nature07991 PMC3969859

[R97] Stark E, Roux L, Eichler R, Senzai Y, Royer S, and Buzsáki G (2014). Pyramidal cell-interneuron interactions underlie hippocampal ripple oscillations. Neuron 83, 467–480. 25033186 10.1016/j.neuron.2014.06.023 PMC4393648

[R98] Strauss KA, Puffenberger EG, Huentelman MJ, Gottlieb S, Dobrin SE, Parod JM, Stephan DA, and Morton DH (2006). Recessive symptomatic focal epilepsy and mutant contactin-associated protein-like 2. N. Engl. J. Med 354, 1370–1377. 16571880 10.1056/NEJMoa052773

[R99] Suh J, Rivest AJ, Nakashiba T, Tominaga T, and Tonegawa S (2011). Entorhinal cortex layer III input to the hippocampus is crucial for temporal association memory. Science 334, 1415–1420. 22052975 10.1126/science.1210125

[R100] Taylor MJ, Rosenqvist MA, Larsson H, Gillberg C, D’Onofrio BM, Lichtenstein P, and Lundström S (2020). Etiology of Autism Spectrum Disorders and Autistic Traits Over Time. JAMA Psychiatry 77, 936–943. 32374377 10.1001/jamapsychiatry.2020.0680 PMC7203675

[R101] Tort ABL, Komorowski R, Eichenbaum H, and Kopell N (2010). Measuring phase-amplitude coupling between neuronal oscillations of different frequencies. J. Neurophysiol 104, 1195–1210. 20463205 10.1152/jn.00106.2010 PMC2941206

[R102] Turrigiano G (2011). Too many cooks? Intrinsic and synaptic homeostatic mechanisms in cortical circuit refinement. Annu. Rev. Neurosci 34, 89–103. 21438687 10.1146/annurev-neuro-060909-153238

[R103] Vandecasteele M, M, S., Royer S, Belluscio M, Berényi A, Diba K, Fujisawa S, Grosmark A, Mao D, Mizuseki K, (2012). Large-scale recording of neurons by movable silicon probes in behaving rodents. J. Vis. Exp 61, e3568. 10.3791/3568 PMC3399468 22415550

[R104] Varga C, Golshani P, and Soltesz I (2012). Frequency-invariant temporal ordering of interneuronal discharges during hippocampal oscillations in awake mice. Proc. Natl. Acad. Sci. USA 109, E2726–E2734. 23010933 10.1073/pnas.1210929109 PMC3479571

[R105] Vogt D, Cho KKA, Lee AT, Sohal VS, and Rubenstein JLR (2015). The parvalbumin/somatostatin ratio is increased in Pten mutant mice and by human PTEN ASD alleles. Cell Rep 11, 944–956. 25937288 10.1016/j.celrep.2015.04.019 PMC4431948

[R106] Vogt D, Cho KKA, Shelton SM, Paul A, Huang ZJ, Sohal VS, and Rubenstein JLR (2018). Mouse Cntnap2 and Human CNTNAP2 ASD Alleles Cell Autonomously Regulate PV+ Cortical Interneurons. Cereb. Cortex 28, 3868–3879. 29028946 10.1093/cercor/bhx248 PMC6455910

[R107] Walf AA, and Frye CA (2007). The use of the elevated plus maze as an assay of anxiety-related behavior in rodents. Nat. Protoc 2, 322–328. 17406592 10.1038/nprot.2007.44 PMC3623971

[R108] Wu YK, Hengen KB, Turrigiano GG, and Gjorgjieva J (2020). Homeostatic mechanisms regulate distinct aspects of cortical circuit dynamics. Proc. Natl. Acad. Sci. USA 117, 24514–24525. 32917810 10.1073/pnas.1918368117 PMC7533694

[R109] Yamamoto J, and Tonegawa S (2017). Direct Medial Entorhinal Cortex Input to Hippocampal CA1 Is Crucial for Extended Quiet Awake Replay. Neuron 96, 217–227.e4. 28957670 10.1016/j.neuron.2017.09.017 PMC5672552

[R110] Yamamoto J, Suh J, Takeuchi D, and Tonegawa S (2014). Successful execution of working memory linked to synchronized high-frequency gamma oscillations. Cell 157, 845–857. 24768692 10.1016/j.cell.2014.04.009

[R111] Yang M, Silverman JL, and Crawley JN (2011). Automated three-chambered social approach task for mice. Curr. Protoc. Neurosci Chapter 8, 26. 21732314 10.1002/0471142301.ns0826s56 PMC4904775

[R112] Ylinen A, Bragin A, Nádasdy Z, Jandó G, Szabó I, Sik A, and Buzsáki G (1995). Sharp wave-associated high-frequency oscillation (200 Hz) in the intact hippocampus: network and intracellular mechanisms. J. Neurosci 15, 30–46. 7823136 10.1523/JNEUROSCI.15-01-00030.1995 PMC6578299

