# Supplementary material for: Hippocampal gamma and sharp-wave ripple oscillations are altered in a Cntnap2 mouse model of autism spectrum disorder
Source: Cell Rep. Author manuscript; Available in PMC 2022 Jan 22. (PMC8783641; doi:10.1016/j.celrep.2021.109970)
Supplement: 1 [file NIHMS1756251-supplement-1.pdf]

Cell Reports, Volume *37*

Supplemental information

Hippocampal gamma and sharp-wave ripple oscillations are altered in a *Cntnap2* mouse model of autism spectrum disorder

Rosalia Paterno, Joseane Righes Marafiga, Harrison Ramsay, Tina Li, Kathryn A. Salvati, and Scott C. Baraban

Cell Reports, Volume *37*

Supplemental information

Hippocampal gamma and sharp-wave ripple oscillations are altered in a *Cntnap2* mouse model of autism spectrum disorder

Rosalia Paterno, Joseane Righes Marafiga, Harrison Ramsay, Tina Li, Kathryn A. Salvati, and Scott C. Baraban

Supplemental Fig. 1

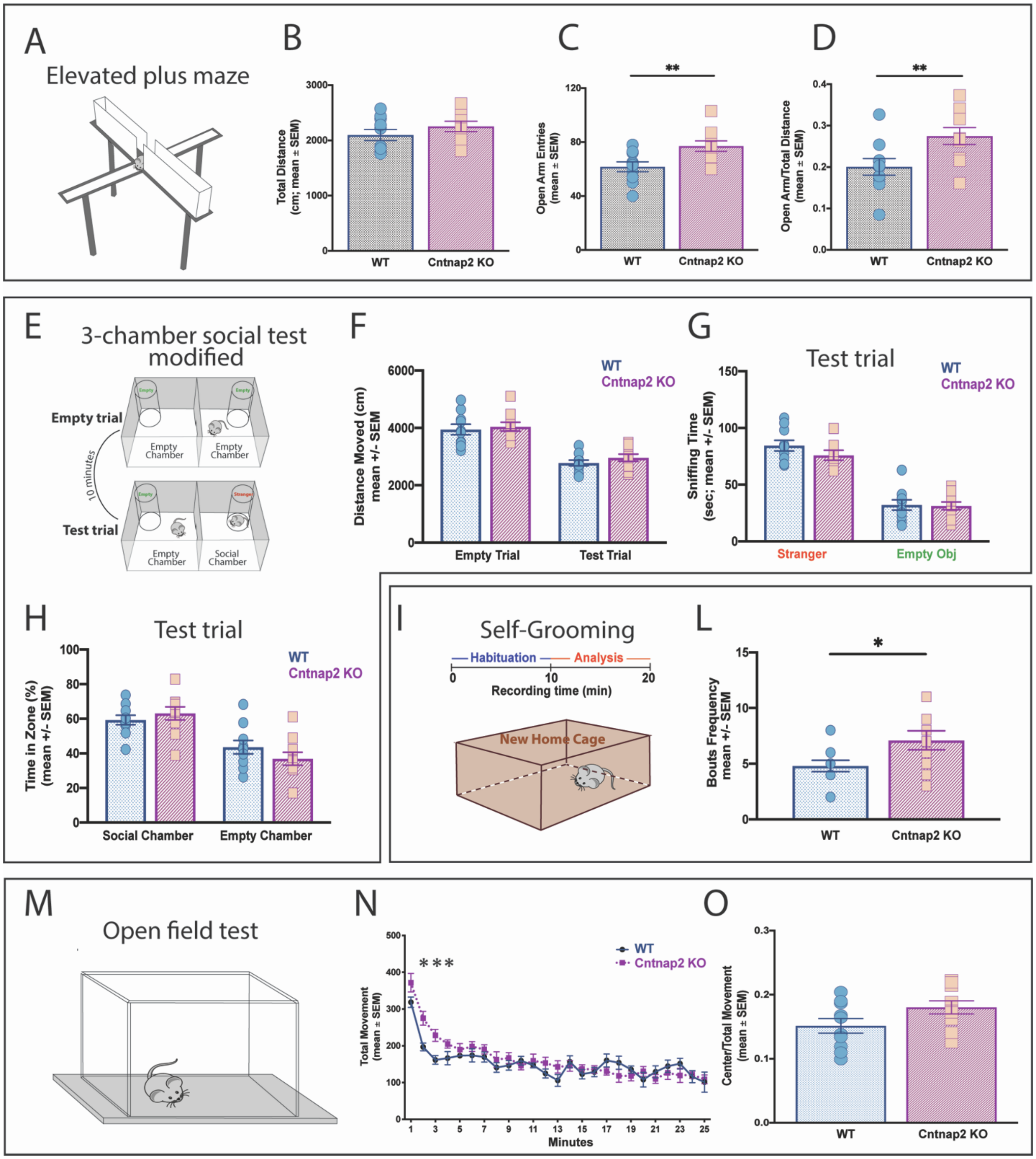

Supplemental Figure 1. ***Cntnap2 KO behavioral phenotype***. (A-D) *Elevated plus maze performance* (WT, n=10; *Cntnap2 KO*, n=10). (A) Elevated plus maze task schematic representation. (B) Total distance (unpaired t test, t (1.1), p>0.05). (C) Number of entries in the open arm (unpaired t test, t (2.9), p<0.01). (D) Total distance in the open arm corrected for the total distance travelled (unpaired t test, t (2.6), p<0.03). Note the increased number of entries and distance travelled in the open arm in *Cntnap2* KO mice compared to aged-matched control. (E-H) *Modified three-chamber social task* (WT, n=10; *Cntnap2 KO*, n=10). (E) Modified three-chamber social task schematic representation. The figure shows the display of the cups during the empty trial and the test trial. The retention interval was 10 minutes. (F) Total distance travelled during the task performance (two-way ANOVA, main effect of trials, p<0.05; main effect of animal condition, p>0.05). (G) Time sniffing the empty cup and the cup with an unfamiliar mouse (two-way ANOVA, main effect of cups, p<0.05; main effect of animal condition, p>0.05). (H) Percentage of time in the zone with the empty cup and the cup with the unfamiliar mouse (two-way ANOVA, main effect of chambers, p<0.05; main effect of animal condition, p>0.05). Note *Cntnap2* KO mice spent more time with the cup containing the unfamiliar mouse. (I-L) *Self grooming* (WT, n=10; *Cntnap2 KO*, n=10). (I) Schematic representation of the self-grooming task in which each animal was recorded for 20 minutes in the new home cage and the analysis was restricted to the last 10 minutes. (L) Frequency of self-grooming bouts (unpaired t test, t (2.3), p<0.05). Note the increased number of self-grooming bouts in *Cntnap2* KO mice. (M-O) *Open field task* (WT, n=10; *Cntnap2 KO*, n=10). (M) schematic representation of the open field task with clear walls. (N) Total distance moved across time (ANOVA p<0.05; Sidak's multiple post-hoc comparisons test; significant time points labeled with *). (O) Movement in the center of the field versus the total number of movements (unpaired t test, t (1.9), p>0.05). Note the increased locomotor activity in *Cntnap2* KO mice. Results are expressed as mean ± SEM. * p<0.05. WT: wild type. Related to Figure 3.

Supplemental Fig. 2

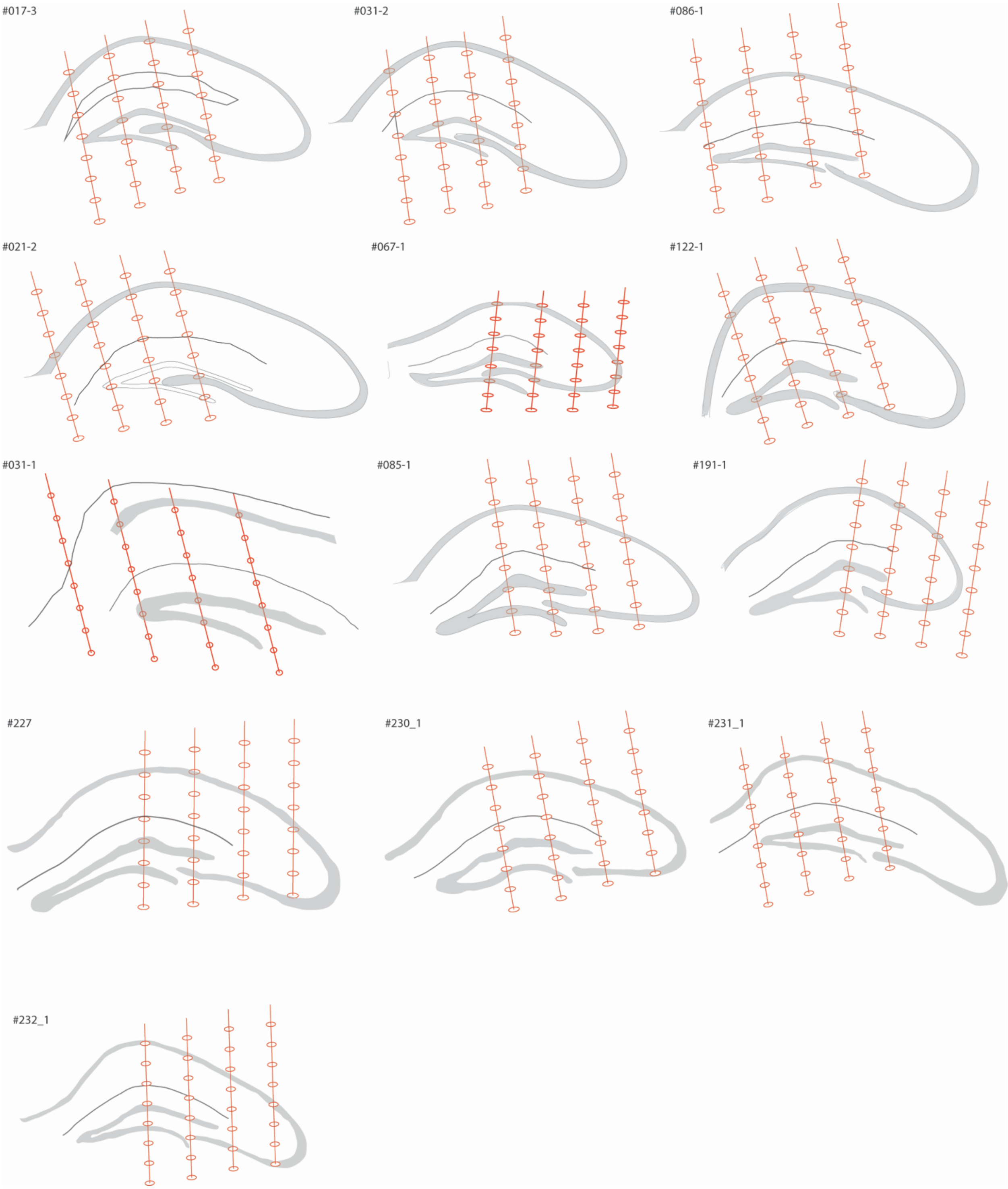

Supplemental Figure 2. ***Schematic representation of the 32-channel silicon probe in the hippocampus of each animal used in this study***. Related to Figure 4.

Supplemental Fig. 3

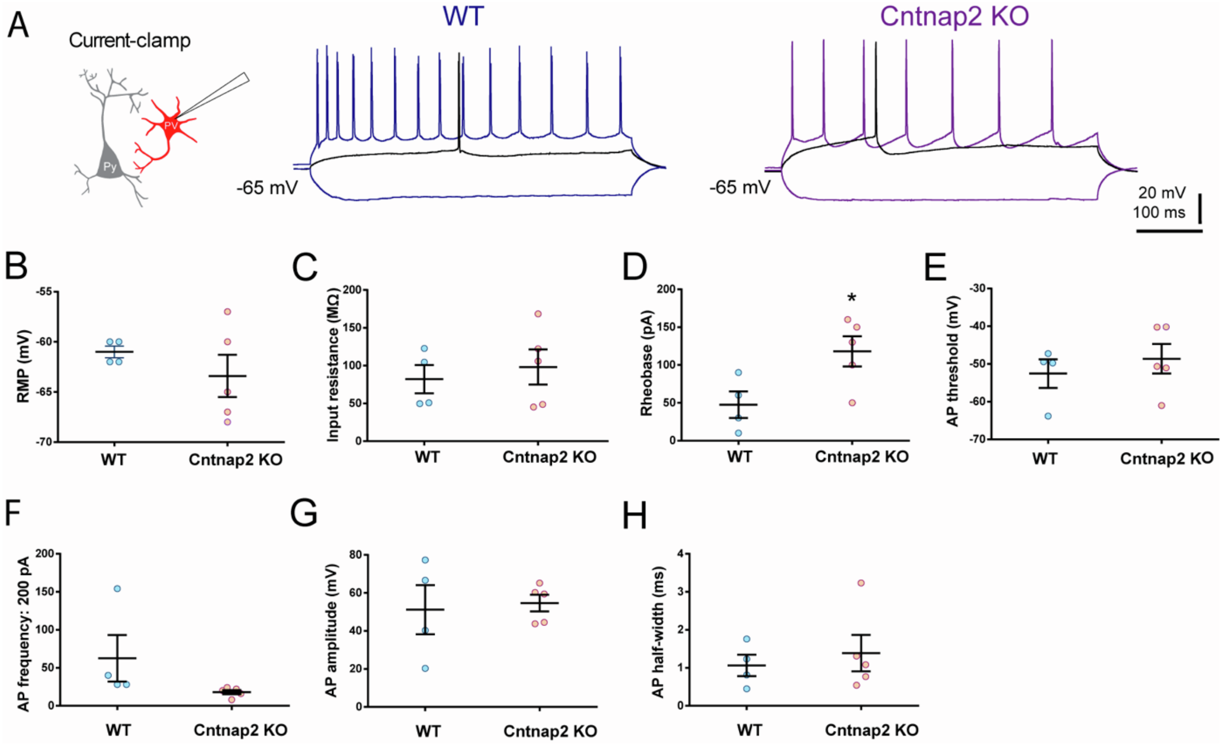

Supplemental Figure 3. ***Intrinsic properties of hippocampal PV+ interneurons in WT and Cntnap2 KO mice***. (A) Whole-cell current-clamp recordings of WT (left) and *Cntnap2* KO (right) PV+ interneurons at 60 days (WT n=4, 2 mice; *Cntnap2 KO* n=5, 3 mice) showing the depolarization step corresponding to the AP threshold (black traces), the maximal firing rate (+150pA/500ms) and the hyperpolarization step (-150 pA/500ms) (blue for WT, purple for *Cntnap2* KO). (B) RMP, (C) Input resistance, (D) Rheobase, (E) AP threshold, (F) AP frequency, (G) AP amplitude and (H) AP half-width. Note that *Cntnap2* KO PV+ interneurons showed an increased Rheobase (*P=0.036); no significant differences were identified in the other parameters (p>0.05). Data are presented as mean ± SEM. Unpaired t test. RMP: Resting membrane potential, AP: action potential, WT: wild type. Related to Figure 2.

Supplemental Fig. 4

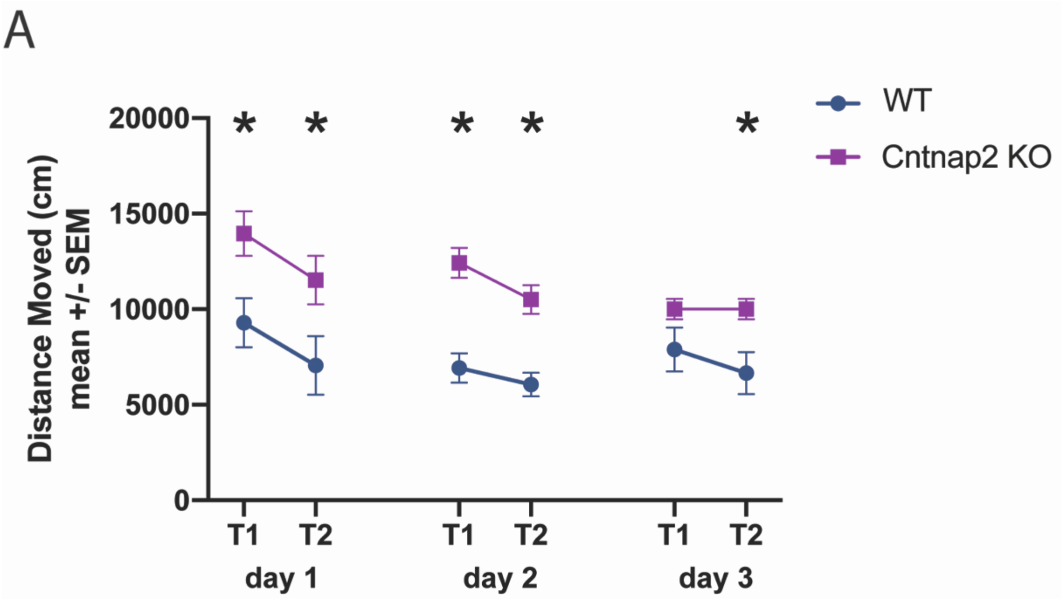

Supplemental Figure 3. ***Open field task performance during in vivo recording***. (A) Distance moved during exposure in the open fields across days. T1 represents the first exposure of the day in the open field and T2 represents the second exposure after a period of rest in the home cage. Note the presence of hyperactivity in *Cntnap2* KO mice. Results are expressed as mean ± SEM. Unpaired t test, * $p<0.05$. Related to Figure 4.

Supplemental Fig. 5

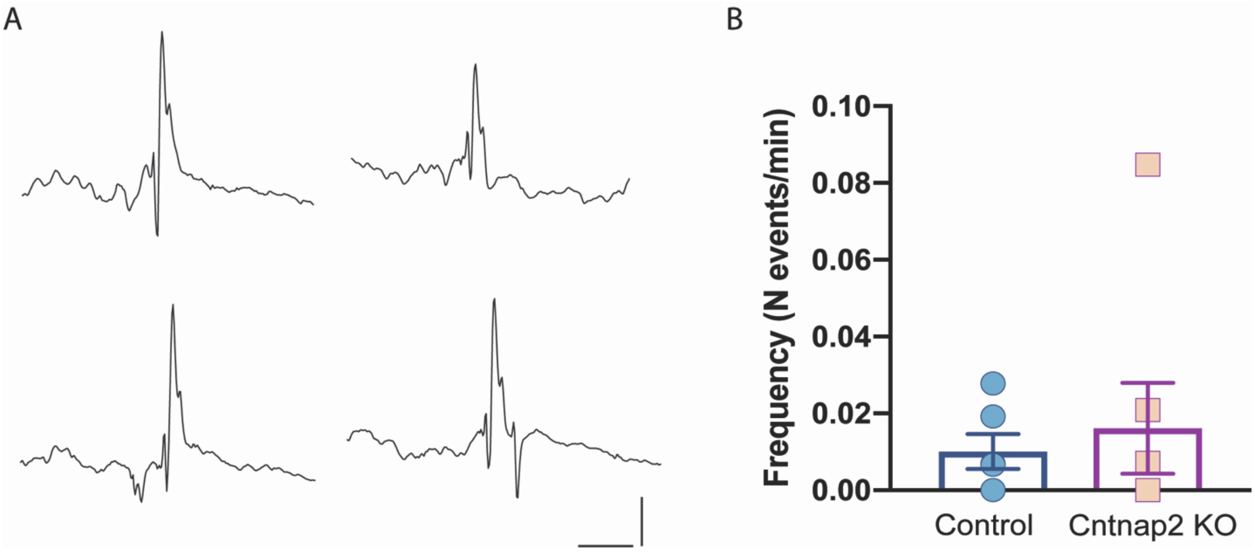

Supplemental Figure 5. Interictal epileptiform discharge (IEDs). (A) Sample IEDs from a Cntnap2 KO mouse (scale bar = 500 µV, 100 ms). (B) IEDs incidence (WT, 6; Cntnap2 KO, 7; unpaired t test, t (1.5), $p > 0.05$). Data are represented as mean ± SEM. Circles represent each animal. WT: wild type. Related to Figure 7.

Suppl. Table 1. Kinetics of sIPSC in CA1 pyramidal neurons from WT and *Cntnap2* KO mice.

Group	Cells (n)	Amplitude (pA)	Frequency (Hz)	Decay time constant (ms)	10-90% Rise-time (ms)
**WT**	40-42	23.58±1.39	6.10±0.40	2.39±.0.12	3.11±0.14
**Cntnap2 KO**	38	16.22±1.09**	4.53±0.29*	2.08±0.14	2.86±.0.11

Data are expressed as mean ± SEM. Unpaired t test, * $p<0.01$; ** $p< 0.0001$. Related to Figure 2.

Suppl. Table 2. Intrinsic firing properties of CA1 PV+ interneurons in WT and *Cntnap2 KO* mice.

Group	Cells (n)	RMP (mV)	Rin (MΩ)	Rheobase (pA)	AP threshold (mV)	AP frequency: 150pA	AP amplitude (mV)	AP Half-width (ms)
**WT**	4	-61.00±0.57	82.03±18.66	47.50±17.50	-52.57±3.79	62.50±30.63	51.15±12.88	1.06±0.28
**Cntnap2 KO**	5	-63.40±2.11	98.13±23.24	118.0±19.85*	-48.63±3.90	18.00±2.82	54.63±4.39	1.38±0.47

Data are expressed as mean ± SEM. Unpaired t test, * $p<0.05$ when compared with the control group. RMP: resting membrane potential; Rin: input resistance; AP: action potential. Related to Figure 2.
